# Specific prostaglandins are produced in the migratory cells and the surrounding substrate to promote *Drosophila* border cell migration

**DOI:** 10.3389/fcell.2023.1257751

**Published:** 2024-01-12

**Authors:** Samuel Q. Mellentine, Hunter N. Brown, Anna S. Ramsey, Jie Li, Tina L. Tootle

**Affiliations:** ^1^ Anatomy and Cell Biology, University of Iowa Carver College of Medicine, Iowa City, IA, United States; ^2^ Biology, University of Iowa, Iowa City, IA, United States

**Keywords:** prostaglandins, cell migration, border cells, *Drosophila*, PGE_2_, PGF_2α_, integrins, myosin

## Abstract

**Introduction:** A key regulator of collective cell migration is prostaglandin (PG) signaling. However, it remains largely unclear whether PGs act within the migratory cells or their microenvironment to promote migration. Here we use *Drosophila* border cell migration as a model to uncover the cell-specific roles of two PGs in collective migration. The border cells undergo a collective and invasive migration between the nurse cells; thus, the nurse cells are the substrate and microenvironment for the border cells. Prior work found PG signaling is required for on-time border cell migration and cluster cohesion.

**Methods:** Confocal microscopy and quantitative image analyses of available mutant alleles and RNAi lines were used to define the roles of the PGE_2_ and PGF_2α_ synthases in border cell migration.

**Results:** We find that the PGE_2_ synthase cPGES is required in the substrate, while the PGF_2α_ synthase Akr1B is required in the border cells for on-time migration. Akr1B acts in both the border cells and their substrate to regulate cluster cohesion. One means by which Akr1B may regulate border cell migration and/or cluster cohesion is by promoting integrin-based adhesions. Additionally, Akr1B limits myosin activity, and thereby cellular stiffness, in the border cells, whereas cPGES limits myosin activity in both the border cells and their substrate. Decreasing myosin activity overcomes the migration delays in both *akr1B* and *cPGES* mutants, indicating the changes in cellular stiffness contribute to the migration defects.

**Discussion:** Together these data reveal that two PGs, PGE_2_ and PGF_2α_, produced in different locations, play key roles in promoting border cell migration. These PGs likely have similar migratory versus microenvironment roles in other collective cell migrations.

## Introduction

Coordinated migration of groups of cells, termed collective cell migration, drives development and tissue repair, and is co-opted during cancer metastasis (Friedl and Gilmour, 2009; Scarpa and Mayor, 2016). Such migrations are regulated by factors from both the migrating cells and their microenvironment (Fife et al., 2014; Kai et al., 2016; Stuelten et al., 2018). One means of regulating collective migration is prostaglandin (PG) signaling (Menter and Dubois, 2012; Tootle, 2013; Kobayashi et al., 2018). PGs are short-range lipid signaling molecules (Funk, 2001; Tootle, 2013). PG signaling begins with cyclooxygenase (COX) enzymes converting arachidonic acid into the prostaglandin precursor (PGH_2_), which is used by PG-type specific synthases to produce bioactive PGs (PGI_2_, PGE_2_, PGF_2α_, PGD_2_ and TXA_2_). These PGs signal in an autocrine or paracrine fashion to activate G-protein coupled receptors (GPCRs) and downstream signaling.

PGs promote collective migration. In zebrafish, global loss of PG signaling impairs migration and delays gastrulation (Cha et al., 2005; Cha et al., 2006). Exogenous application of PGs to cultured cancer cells increases motility. In patients, inhibition of PG synthesis via COX inhibitors reduces the risk of cancer metastasis (Li et al., 2012; Menter and Dubois, 2012). While these studies established PG signaling promotes migration, it remains unclear which PG or PGs regulate migration, whether PGs act within the migrating cells and/or their microenvironment, and how specific PGs drive migration.

**FIGURE 1:**
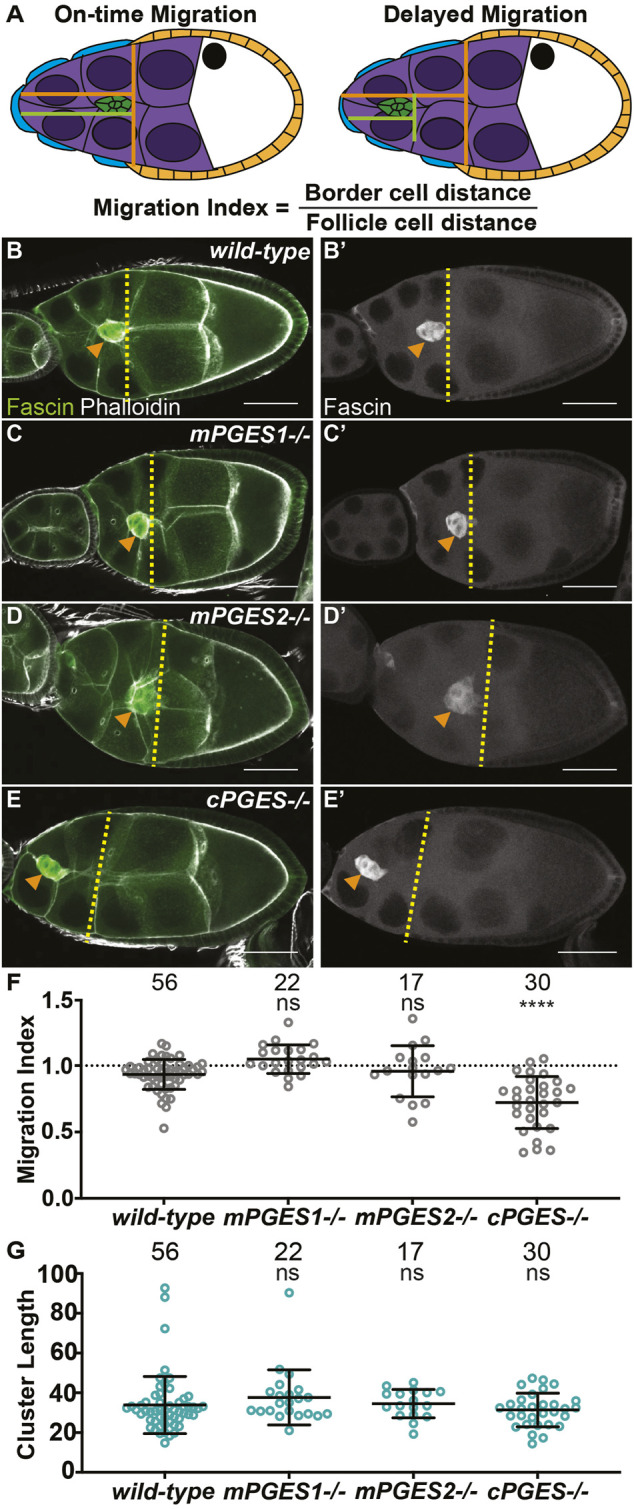
cPGES is required for on-time border cell migration. **(A)**. Schematic of on-time (left) and delayed (right) border cell migration and how the migration index is calculated; anterior is to the left and posterior is to the right. The border cell cluster (green), outer follicle cells (orange), stretch follicle cells (blue), nurse cells (purple), and oocyte (white) are diagramed. **(B‐E’)**. Maximum projections of 3 confocal slices of S9 follicles stained for Fascin (green in merge) and F-actin (phalloidin, white in merge). Orange arrowheads indicate the border cell cluster and yellow dashed lines indicate the position of the outer follicle cells. Images brightened by 30% to increase clarity. Scale bars = 50 μm. **(B-B’)**. *wild-type* (*yw*). **(C,C’)**. *mPGES1*−/− (*mPGES1* ^*KG04713/KG04713*^). **(D,D’)**. *mPGES2−/−* (*mPGES2* ^*EY13245/EY13245*^). **(E,E’)**. *cPGES−/−* (*cPGES* ^*EY05607/EY05607*^). **(F-G)**. Graphs of migration index **(F)** and border cell cluster length **(G)** for the indicated genotypes. Circle = single follicle; *n* = number of follicles. In **(F)**, the dotted line indicates on-time border cell migration. For **(F,G)**, lines = averages and error bars = SD. ns > 0.05, **** *p* < 0.0001, unpaired *t*-test, two-tailed. In wild-type follicles, throughout S9, the migrating border cell cluster is in-line with the outer follicle cells (**A**, left). When migration is delayed, the cluster remains anterior to the follicle cells (**A**, right). We take advantage of this coordination to calculate the migration index **(A)**, which is the distance of the border cell cluster divided by the distance of the outer follicle cells. On-time migration results in a migration index of ∼1, while delayed migration is < 1. Like wild-type **(B,B’)**, loss of mPGES1 or mPGES2 exhibit on-time border cell migration **(C–D’, F)**, whereas loss of cPGES delays migration **(E–F)**. Border cell cluster length is unaffected by loss of mPGES1, mPGES2 or cPGES **(G)**.

To address these questions, we use the *in vivo*, collective migration of the border cells during *Drosophila* oogenesis. Each ovary contains 16–20 ovarioles, chains of sequentially developing egg chambers or follicles (Giedt and Tootle, 2023). There are 14 stages of follicle development and each follicle is comprised of 16 germline cells–15 nurse cells and one oocyte–and ∼650 somatic cells termed follicle cells. During Stage 9 (S9), 6–8 follicle cells are specified as the border cells, delaminate from the follicular epithelium and migrate posteriorly between the nurse cells to the oocyte (Montell, 2003; Montell et al., 2012). Thus, the nurse cells comprise both the microenvironment and the substrate for border cell migration. Throughout the migration, the border cell cluster is in line with the position of the outer follicle cells, providing an internal control for on-time migration (Figure 1A).

Border cell migration requires PG signaling (Fox et al., 2020). *Drosophila* have a single COX-like enzyme, Pxt (Tootle and Spradling, 2008); subsequently referred to as dCOX1. Loss of dCOX1 both delays migration and elongates the cluster, indicative of defective cohesion. Cell-specific RNAi experiments reveal strong knockdown of dCOX1 in the border cells delays migration and causes cluster compaction, whereas mild knockdown in the nurse cells (i.e., the substrate) decreases cluster cohesion (Fox et al., 2020). These results indicate PGs are produced in both the migratory cells and their substrate to regulate migration, and PGs from the different cell types may control distinct aspects of border cell migration, on-time migration and cluster cohesion.

We sought to determine which PG or PGs are produced in the border cells and/or the substrate, and the downstream mechanisms whereby they promote on-time migration and maintain cluster cohesion. We find that PGE_2_ produced in the nurse cells by the cytosolic PGE_2_ synthase (cPGES), *Drosophila* p23, is required for on-time migration. Whereas, PGF_2α_ produced in the border cells by Akr1B promotes migration. PGE_2_ signaling does not impact cluster cohesion, but PGF_2α_ signaling has distinct cell-specific roles. Knockdown of Akr1B in the border cells results in compacted clusters, whereas knockdown in the substrate causes cluster elongation. The migration delays and/or cluster morphology changes are resolved by S10A in both the *cPGES* and *akr1B* mutants. One potential means by which these PGs may promote border cell migration is by regulating integrins. Integrins are critical cell adhesion factors (Huttenlocher and Horwitz, 2011) and are required for both on-time border cell migration and maintaining cluster cohesion (Dinkins et al., 2008; Llense and Martin-Blanco, 2008). Previously, we found dCOX1 is required for integrin localization to the surface of the border cell cluster (Fox et al., 2020). Here we find that Akr1B is required for integrin localization. Another means of controlling migration is by regulating the balance of forces between the migrating border cells and their substrate, the nurse cells (Majumder et al., 2012; Aranjuez et al., 2016). This mechanoreciprocity is mediated at the cellular level by the activation of non-muscle myosin II (subsequently referred to as myosin). We find that cPGES is required to limit myosin activity in both the border cells and the substrate, whereas Akr1B only limits it within the border cells. Pharmacologically reducing myosin activity restores on-time migration in both the *cPGES* and *akr1B* mutants. These data lead to the model that Akr1B produces PGF_2α_, primarily within the border cells, to regulate both integrin localization and myosin activity within the border cells. In the substrate, cPGES produces PGE_2_, which regulates myosin activity within the substrate and the border cells. Ultimately, the synthesis of both PGE_2_ and PGF_2α_ is required for on-time border cell migration, while only PGF_2α_ modulates cluster cohesion. This work provides the first evidence that multiple types of PGs, produced from different cellular sources, work in concert to control collective cell migration by both overlapping and distinct mechanisms. Given the conservation of PG signaling, such a multi-cellular and multi-PG mechanism of promoting cell migration is likely conserved across organisms and tissues.

## Materials and methods

### Reagents and resources

See Supplementary Table S1 for detailed information on the reagents used in these studies and Supplementary Table S2 for the specific genotypes used in each figure panel. All raw data used in this study can be found in Supplementary Table S3.

### Fly stocks

Fly stocks were maintained on cornmeal/agar/yeast food at 21°C, except where noted. Before immunofluorescence staining, newly eclosed flies were fed wet yeast paste every day for 2–4 days. Unless specified, *yw* (BDSC 1495) was used as the control. The following stocks were obtained from the Bloomington *Drosophila* Stock Center: *c355 GAL4* (BDSC 3750), *actin-5C GAL4* (BDSC 8807), *mgst1* ^KG04713^ (BDSC 13839), *Su(P)* ^*EY13245*^ (BDSC 20866)*, p23* ^*EY05607*^(BDSC 16661)*, akr1B* ^*PL00034*^ (BDSC 19594)*, akr1B* ^*EY07011*^ (BDSC 16777), *p23* RNAi HMJ24151 (BDSC 62911), *p23* RNAi-2 GL01292 (BDSC 41862), *akr1B* deficiency-1 DF(3L)BSC577 (BDSC 25411), *akr1B* deficiency-2 DF(3L)ED4475 (BDSC 8069), *akr1B* RNAi HMS05657 (BDSC 67838), *akr1B* RNAi-2 HMC05226 (BDSC 62219) and UAS Dicer 2 (BDSC 24651). The following stocks were obtained from the Exelexis Stock Center: *mgst1* ^d10243^, *akr1B* ^d00405^ and *pxt* ^*f01000*^ (Thibault et al., 2004). The *oskar GAL4* line (second chromosome; BDSC 44241) was a generous gift from Anne Ephrussi [European Molecular Biology Laboratory; (Telley et al., 2012)]. Expression of the RNAi lines were achieved by crossing to *actin-5C GAL4, c355 GAL4* or *oskar GAL4*, maintaining fly crosses at 21°C and maintaining progeny at 29°C for 5–6 days. *UAS Dicer* was used in combination with *c355* to enhance RNAi efficiency where noted in the figure legends.

### Immunofluorescence

*Drosophila* ovaries (5-8 pairs per sample) were dissected into room temperature Grace’s insect medium (Lonza). Ovaries were fixed for 10 min using 4% paraformaldehyde diluted in Grace’s medium. Samples were washed six times for 10 min each at room temperature in antibody wash (1X phosphate-buffered saline [PBS], 0.1% Triton X and 0.1% bovine serum albumin [BSA]). Primary antibodies were diluted in antibody wash and incubated overnight at 4°C, except for β_PS_-integrin which was incubated for ∼48–72 h at 4°C. The following monoclonal antibodies were obtained from the Developmental Studies Hybridoma Bank (DSHB), created by the NICHD of the NIH and maintained at The University of Iowa, Department of Biology, Iowa City, IA: mouse anti-Fascin 1:50 [sn7c, Cooley, L; AB_528239 (Kelly Cant et al., 1994)], mouse anti-β_PS_-integrin 1:10 [CF.6G11, Brower, D; AB_528310 (Danny et al., 1984)], and mouse anti-EYA 1:100 [eya 10H6 (Boyle et al., 1997)]. Rabbit polyclonal antibodies (Genscript) produced against full-length *Drosophila* p23 (FBpp0300672) and full-length *Drosophila* Akr1B (FBpp0303936) was used at 1:1000. After six washes in antibody wash (10 min each), samples were incubated in secondary antibodies overnight at 4°C. The following secondaries were used at 1:500: AF488∷goat anti-mouse (AB_2534069), AF568∷ goat anti-mouse (AB_2534072), AF488 goat anti-rabbit (AB_2576217), and AF568∷ goat anti-rabbit (AB_ 2534102; Thermo Fischer Scientific). Alexa Fluor 586- or Alexa Fluor 647-conjugated phalloidin (A12380 and A22287; Thermo Fischer Scientific) diluted 1:250 were included in both primary and secondary antibody incubations. Following six washes in antibody wash (10 min each), 4′,6-diamidino-2-phenylidole (DAPI; 5 mg/mL; D3571; Thermo Fischer Scientific) staining was performed at a concentration of 1:5000 in 1X PBS for 10 min at room temperature. Samples were then rinsed in 1X PBS and mounted on slides in 1 mg/mL phenylenediamine in 50% glycerol, pH 9 (Platt and Michael, 1983). All experiments were performed a minimum of three independent times.

Phospho-myosin regulator light chain (pMRLC) staining was performed using a protocol provided by the McDonald Lab (Majumder et al., 2012; Aranjuez et al., 2016). Briefly, ovaries were fixed for 20 min at room temperature in 8% paraformaldehyde in 1x PBS and 0.5% Triton X-100. Samples were blocked for 30 min at room temperature in Triton antibody wash (1X PBS, 0.5% Triton X-100, and 5% BSA). Primary antibodies, rabbit anti-pMRLC (S19, 1:100; AB_330248; Cell Signaling), mouse anti-Hts [1:50; Lipshitz, H; DSHB; AB_ 528070; (Zaccai and Lipshitz, 1996)] and mouse anti-FasIII [1:50, Goodman, C; DSHB; AB_ 528238; (Patel et al., 1987)], were diluted in Triton antibody wash and incubated for ∼48–72 h at 4°C. Following six washes in Triton antibody wash (10 min each), secondary antibody staining, washes, and DAPI staining were performed, and samples were mounted as described above.

For the myosin pharmacological inhibition studies, *Drosophila* ovaries were dissected in Stage 9 (S9) medium (Prasad and Montell, 2007). S9 media consists of Schneider’s medium (Sigma-Aldrich, SCR_008988), 0.6 x penicillin/streptomyocin (Life Technologies, SCR_008817), 0.2 mg/mL insulin (Sigma-Aldrich, SCR_008988), and 15% fetal bovine serum (Atlanta Biologicals). Ovaries were teased apart and incubated at room temperature for 2 h in control medium or 200 μM of Y-27632 (Y0503, Millipore Sigma, SCR_2298772). After 2 h, ovaries were rinsed with S9 medium and fixed and stained for mouse anti-Fascin 1:50 [sn7c, Cooley, L; AB_528239; (Kelly Cant et al., 1994)], phalloidin, and DAPI using the protocol for pMRLC staining described above.

### Image acquisition and processing

Microscope images for fixed and stained *Drosophila* follicles were taken using LAS SPE Core software on a Leica TCS SPE mounted on a Leica DM2500 using ACS APO 20x/0.6 IMM Corr -/D objective (Leica Microsystems), LAS-X software (SCR_013673) on a Leica DMi8 Stellaris using a HCPLAPO CS2 20x/0.75 Dry and HCPL APO CS2 63x/1.4 Oil, Zen software (SCR_013672) on Zeiss 700 LSM mounted on an Axio Observer.Z1 using a Plan-Apochromat 20x/0.8 M27 or EC-Plan_Neo_Fluar 40x/1.3 Oil, Zeiss 880 mounted on Zeiss Axio Observer.Z1 using Plan-Apochromat 20x/0.8, Plan-Apochromat 40x/1.3 oil (Carl Zeiss Microscopy), or NIS-Elements Software (SCR_014329) on Nikon ECLIPSE Ti2-E inverted microscope using Plan Apo λD 20x/0.8 Dry. S9 follicles were identified by their size (∼150 μm–250 μm) and morphology, including, the location of the outer follicle cells and the border cell cluster. The beginning of S10A was defined as when the anterior most outer follicle cells reached the nurse cell-oocyte boundary and flattened. Maximum projections, merge, rotation, and cropping were performed using ImageJ software (FIJI, RRID: SCR 002285 (Abramoff et al., 2004)). All images shown were brightened by 30% in Photoshop (Adobe, RRID: SCR 014199), except where noted, to improve visualization and figures were made using Illustrator (Adobe, RRID: SCR 010279).

### Quantification of border cell migration and cluster length

Quantification of the Migration Index (MI) was performed as described previously (Fox et al., 2020; Lamb et al., 2020). Briefly, measurements of S9 follicles were performed using ImageJ software (Abramoff et al., 2004) on maximum projections of 2-4 confocal slices of follicles stained for Fascin and phalloidin. A line segment was used to measure the distance in microns from the anterior end of the follicle to the leading edge of the border cell cluster; this was defined as the border cell distance. Another line segment was used to measure the distance from the anterior end of the follicle to the anterior end of the outer follicle cells: this was defined as the outer follicle cell distance. The entire follicle length was also measured along the anterior-posterior axis. The migration index was calculated by dividing the border cell distance by the follicle cell distance. Cluster length was determined by measuring the distance from the front to the rear of the border cell cluster (detached cells were not included). Data were compiled in and calculations were performed in Excel (Microsoft, RRID: SCR 016137), and graphs were generated and statistical analyses performed using Prism (GraphPad Software, RRID SCR 002798).

Stage 10A (S10A) analyses of migration completion and number of border cells was performed as described previously (Fox et al., 2020). Briefly, quantifications of S10A follicles were performed using ImageJ software (Abramoff et al., 2004) on maximum projections of 2-4 confocal slices of follicles stained for: Fascin for assessing completion of migration, and either DAPI or EYA for quantifying the number of border cells in the cluster and the number left along the migration path. Data were compiled in and calculations were performed in Excel (Microsoft, RRID: SCR 016137), and graphs were generated and statistical analyses performed using Prism (GraphPad Software, RRID SCR 002798).

### Quantification of integrin localization

Integrin analysis was performed using the method described previously (Fox et al., 2020). Briefly, integrin intensity was measured from single confocal slices of immunofluorescence images of fixed *Drosophila* follicles. The “straight line” function was used to draw lines between 4–7 microns at three different locations on the border cell membranes, and highest fluorescence intensity value was measured for β_PS_-integrin and phalloidin. The integrin value was divided by the phalloidin value, the average was calculated for the three segments, and then the average was normalized to the overall *wild-type* average*.* Data were compiled in and calculations were performed in Excel, and graphs were generated and statistical analyses performed using Prism.

### pMRLC quantifications

pMRLC analysis was performed as previously described (Lamb et al., 2021). Briefly, intensity measurements were performed on maximum projections of 3 confocal slices of 40x confocal images at a 2x zoom using ImageJ software ((Abramoff et al., 2004). The fluorescence intensity of pMRLC on the border cells was measured by tracing the shape of the border cell cluster and obtaining the highest intensity value of the pMRLC staining. The same shape was used to measure the background within the substrate, which was substracted. pMRLC values were normalized to the *wild-type* average. To determine the substrate pMRLC intensity, three-line segments (14–16 μm) at different locations within the follicle were used to measure both pMRLC and phalloidin fluorescent intensity peaks. Values were normalized by division with the phalloidin intensities and averaged for one sample. Averages were then normalized to the *wild-type* average. Data was compiled in and calculations were performed in Excel, and graphs were generated and statistical analyses performed using Prism.

### Western blot

Whole ovary pairs (5 total per sample) were dissected at room temperature in 1xPBS and transferred to a 1.5 mL tube containing 80 μL 1xPBS. 20 μL 5x Laemmli buffer was added and lysis was performed by grinding tissue with plastic pestles (RNase-free disposable pellet pestles; Thermo Scientific). Samples were boiled for 10 min and briefly spun down before loading. Western Blots were performed using standard methods. Briefly, samples were run on 10% SDS-PAGE gels, and transferred onto nitrocellulose membranes (Amersham Protran 0.2 μm NC; GE Healthcare Life Sciences). Ladders used were either Precision Plus Protein All Blue Standards (161-0373; Bio-Rad Laboratories) and Precision Plus Protein Dual Color Standards (161-0374; Bio-Rad Laboratories). In some cases, blots were cut horizontally prior to primary antibody incubation to allow for the assessment of two different proteins. Blots were washed three times in 1X Tris-Buffered Saline (TBS) and once in 1X TBS with 1% Tween 20 (TBST) for 10 min each. The following antibodies and concentrations were used: rabbit anti-p23 at 1:50,000 (produced to full-length *Drosophila* p23 (FBpp0300672) by GenScript), rabbit anti-Akr1B-2 at 1:100,000 (produced to full-length *Drosophila* Akr1B (FBpp0303936) by GenScript) and mouse anti-α-Tubulin at 1:5000 (AB_477593; Sigma-Aldrich). Antibodies were diluted in Western blot block (5% non-fat dry milk in TBST) and incubated over-night at 4°C. Blots were washed four times with TBS and once with TBST, for 10 min each. The following secondaries were used at 1:5000 in 5 mL Western blot block: Peroxidase-AffiniPure Goat Anti Rabbit IgG (H + L) and Peroxidase-AffiniPure Goat Anti Mouse IgG (H + L) (Jackson ImmunoResearch Laboratories). Blots were washed four times with TBS and twice with TBST, for 10 min each. Blots were developed with SuperSignal West Pico Chemiluminescent Substrate or SuperSignal West Femto Maximum Sensitivity Substrate (SCR_008452; Thermo Scientific). Blots were imaged and analyzed on the Amersham Imager 600 series Scanning Chemiluminescence (GE Healthcare Life Sciences). Data were collected and analyzed in Excel and graphs were generated and statistical analyses performed in Prism.

## Results

### cPGES is required for on-time border cell migration

Loss of all PG synthesis delays border cell migration and elongates clusters (Fox et al., 2020), however, which specific PGs are involved remains unknown. We first assessed the role of PGE_2_. There are three PGE_2_ synthases, microsomal PGES1 (mPGES1), mPGES2, and cPGES (Jakobsson et al., 1999; Tanioka et al., 2000; Tanikawa et al., 2002). *Drosophila* mPGES1 is encoded by *mgst1* and is 61% similar at the protein level to its human homolog (UniProt O14684), mPGES2 is encoded by *Su(P)* and is 52% similar (UniProt Q9H7Z7), and cPGES is encoded by *p23* and is 45% similar (UniProt Q15185).

Using available insertional alleles, we assessed the roles of these PGE_2_ synthases in border cell migration. In wild-type follicles, the border cell cluster is in-line with the outer follicle cells throughout S9 (Figures 1B, B’), indicating on-time migration. The tested mutations in *mPGES1* and *mPGES2* do not impact border cell migration (Figures 1C–D’), whereas mutation of *cPGES* delays border cell migration as the border cell cluster is anterior to the outer follicle cells (Figures 1E, E’). To quantify border cell migration during S9, we measure the distance of the border cell cluster from the anterior end of the follicle and divide it by the distance of the outer follicle cells; we call this the migration index or MI (Fox et al., 2020; Lamb et al., 2020). On-time migration results in MI of ∼1, whereas delayed migration is < 1 (Figure 1A). Using this method, the MIs in *mPGES1* (1.049, *p* = 0.212) and *mPGES2* (0.958, *p* = 0.529) mutants are similar to wild-type (0.934, Figure 1F). Conversely, loss of cPGES decreases the MI (Figure 1F, 0.722, *p* < 0.0001). These data indicate that cPGES-dependent production of PGE_2_ is required for on-time border cell migration.

We next assessed the roles of the synthases in cluster cohesion. Reduction or loss of cluster cohesion results in elongated clusters (along the anterior/posterior axis) and/or cells detaching from the cluster and being left along the migration path(Niewiadomska et al., 1999; Cai et al., 2014). Indeed, loss of dCOX1 results in both of these defects (Fox et al., 2020). Cluster length was not altered in *mPGES1*, *mPGES2* or *cPGES* mutants (Figure 1G), suggesting that the cluster elongation phenotype observation when all PG synthesis is lost is not due to the loss of PGE_2_ production.

Based on the robust border cell migration delay observed, we focused the rest of our PGE_2_ studies on cPGES. As cPGES has not been previously studied in *Drosophila*, we next characterized the insertional allele. First, we compared border cell migration in follicles from *cPGES* heterozygotes and homozygotes. Surprisingly, heterozygosity for *cPGES* delays border cell migration (Supplementary Figure S1A, MI = 0.717, *p* < 0.01). To uncover why the allele has a dominant phenotype, we developed an antibody to cPGES and quantified protein levels by Western blot analyses. The allele is a loss of function, as homozygosity for the *cPGES* mutation exhibits 23% of wild-type protein levels, while heterozygosity results in a 30% reduction in protein (Supplementary Figure S1B). Together, these data suggest that even mild reductions in cPGES are sufficient to decrease PGE_2_ production enough to impair border cell migration.

As loss of dCOX1 not only delays border cell migration but increases border cell number and exhibits border cells remaining along the migration path in S10A follicles (Tootle and Spradling, 2008; Fox et al., 2020) we also assessed whether loss of cPGES results in similar defects. In the *cPGES* mutant, we find that border cell numbers are unchanged and the cluster reaches the oocyte by S10A (Supplementary Figures S2B, D). However, in ∼60% of S10A follicles there are one to three somatic cells between the anterior substrate cells (Supplementary Figures S2B, E). This latter finding was surprising, as we do not see any cluster elongation during S9. In an attempt to determine whether these cells are border cells that have been left behind or whether the cells aberrantly initiated migration at a later time point, we re-examined our S9 images. Specifically, we used the DAPI staining to look for cells detaching from the border cells cluster (and thereby, losing expression of Fascin, the marker used to label the border cells) and remaining along the migration path. We only observe 2 instances of a somatic cell left behind out of 30 S9 follicles. These data lead us to believe that the cells along the migration path at S10A in our *cPGES* mutants did not detach from the border cell cluster. The origin of these cells and the consequence of their presence remains unknown.

### cPGES is required within the substrate for on-time border cell migration

We next assessed the expression and localization of cPGES within S9 follicles. cPGES is cytoplasmic in all cells of the follicle, both germline and somatic (Supplementary Figures S1C, C’). This staining is reduced in *cPGES* mutant follicles (Supplementary Figures S1D, D’). These data indicate that cPGES is present in both the border cells as well as their substrate, suggesting that PGE_2_ could be produced in either or both cell-types to regulate border cell migration.

**FIGURE 2:**
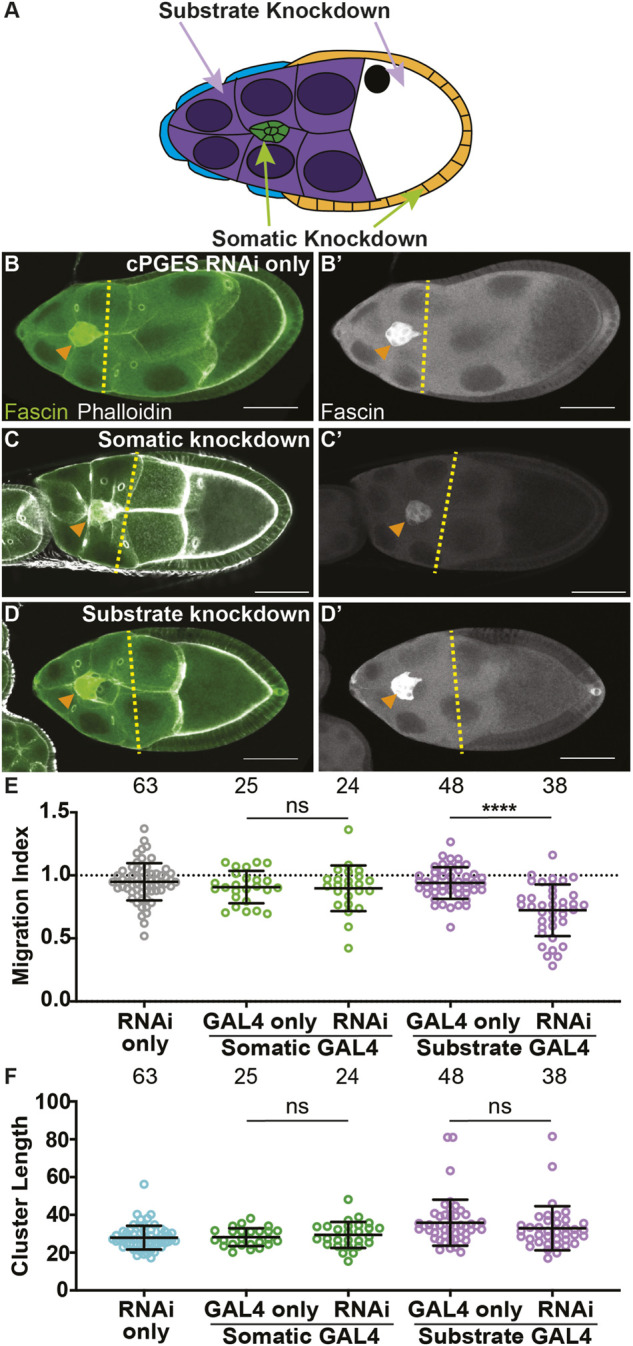
cPGES is required in the substrate for on-time border cell migration. **(A)**. Schematic of a S9 follicle indicating the cell-specific knockdown for each GAL4 driver; somatic knockdown occurs in border cells (green) and follicle cells (blue and orange) and substrate knockdown occurs in the nurse cells (purple). **(B–D’)**. Maximum projections of 3 confocal slices of S9 follicles stained for Fascin (green in merge) and F-actin (phalloidin, white in merge). Orange arrowheads indicate the border cell cluster and yellow dashed lines indicate the position of the outer follicle cells. Images brightened by 30% to increase clarity. Scale bars = 50 μm. **(B,B’)**. cPGES RNAi control (*cPGES RNAi/+*). **(C,C’)**. Somatic knockdown of cPGES (*c355 GAL4/+;* *cPGES RNAi*/+). **(D,D’)**. Substrate knockdown of cPGES (*osk GAL4/cPGES RNAi*). *cPGES RNAi* used was HMJ24151. **(E,F)**. Graphs of migration index **(E)** and border cell cluster length **(F)** for the indicated genotypes. Circle = single follicle; n = number of follicles. In **(E)**, the dotted line indicates on-time border cell migration. For **(E,F)**, lines = averages and error bars = SD. ns > 0.05, **** *p* < 0.0001, unpaired *t*-test, two-tailed. Like the controls **(B-B’,E)**, somatic knockdown of cPGES exhibits on-time border cell migration **(C-C’,E)**, whereas substrate knockdown delays migration **(D‐E)**. Border cell cluster length is unaffected by either somatic or substrate cPGES knockdown **(F)**.

To determine where PGE_2_ synthesis is required for border cell migration, we used the UAS/GAL4 system to knockdown cPGES by RNAi in either all the somatic cells, including the border cells, or the substrate (Figure 2A). We first assessed cPGES function in the somatic cells. As expected, the controls (GAL4 only and RNAi only) exhibit on-time migration (Figures 2B, E; MI = 0.907 and 0.949, respectively). Similarly, somatic knockdown of cPGES does not impact border cell migration (Figures 2C, E; MI = 0.898). Conversely, knockdown of cPGES in the substrate delays border cell migration (Figures 2D, E; MI = 0.722, *p* < 0.0001) compared to the controls (GAL4 and RNAi only; MI = 0.940 and 0.949, respectively). To assess knockdown efficiency, we performed immunofluorescence staining for cPGES. Unlike the controls where cPGES is expressed ubiquitously (Supplementary Figures S3A), somatic cPGES knockdown retains expression in the substrate but has reduced or absent staining within the somatic cells, including the border cells (Supplementary Figures S3B). In the substrate knockdown, cPGES remains expressed in the somatic cells but is reduced in the substrate (Supplementary Figures S3C). The migration results were confirmed using the second RNAi line (RNAi-2, Supplementary Figures S3D). We also assessed the cell-specific roles of cPGES in regulating cluster morphology. Cluster length is normal when cPGES is knocked down in either the somatic cells or the substrate with either RNAi line (Figure 2F; Supplementary Figures S3E). Together, these data reveal cPGES acts within the substrate to promote on-time border cell migration, but has no role in cluster cohesion.

### Akr1B is required for on-time border cell migration

As PG synthesis is required in both the substrate and the migratory cells for migration (Fox et al., 2020), and cPGES acts only in the substrate (Figure 2; Supplementary Figures S3), a different PG must be produced in the border cells to promote migration. We hypothesized it might be PGF_2α_, as PGF_2α_ drives actin remodeling in later stages of *Drosophila* oogenesis (Tootle and Spradling, 2008; Spracklen et al., 2014), and actin dynamics are critical for border cell migration (Montell, 2003; Montell et al., 2012). In mammals, PGF_2α_ is produced by the aldo-keto reductase proteins Akr1B1, Akr1B10, and Akr1C3 (Banerjee, 2021). In *Drosophila*, the most homologous PGF_2α_ synthase is Akr1B; 75% similar to human Akr1B1 (UniProt P15121).

**FIGURE 3:**
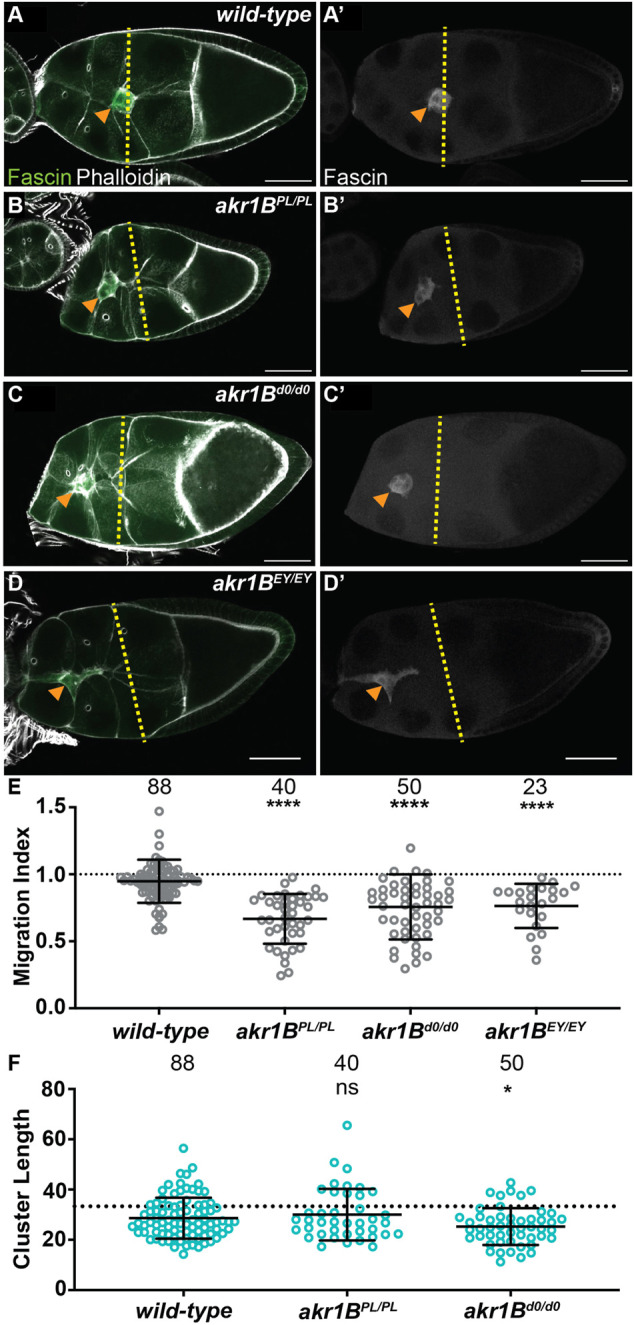
Akr1B is required for border cell migration and cluster morphology. **(A–D’)**. Maximum projections of 3 confocal slices of S9 follicles stained for Fascin (green in merge) and F-actin (phalloidin, white in merge). Orange arrowheads indicate the border cell cluster and yellow dashed lines indicate the position of the outer follicle cells. Images brightened by 30% to increase clarity. Scale bars = 50 μm. **(A,A’)**. *wild-type* (*yw*). **(B,B’)**. *akr1B* ^*PL00034/PL00034*^. **(C,C’)**. *akr1B* ^*d00405/d00405*^. **(D,D’)**. *akr1B* ^*EY07011/EY07011*^. **(E,F)**. Graphs of migration index **(E)** and border cell cluster length **(F)** for the indicated genotypes; n = number of follicles. In **(F)**, the dotted line indicates an on-time border cell migration. For **(E,F)**, lines = averages and error bars = SD. ns > 0.05, *p < 0.05, ** *p* < 0.01 and **** *p* < 0.0001, unpaired *t*-test, two-tailed. In wild-type S9 follicles, the migrating border cells are in-line with the outer follicle cells **(A-A’,E)**, whereas all three *akr1B* alleles result in delayed migration **(B–E)**. The border cell cluster is more compact in *akr1B* ^*d0/d0*^ but not the *akr1B* ^*PL/PL*^ allele **(F)**.

We used three insertional alleles to assess the role of Akr1B in border cell migration. While wild-type follicles exhibit on-time migration (Figures 3A, E; MI = 0.948) all three alleles of *akr1B* (PL*,* d0 and EY) delay migration (Figures 3B–E; MIs = 0.668, 0.645 and 0.757, respectively, *p* < 0.0001). However, the delay in the *akr1B* ^*EY*^ allele was milder, suggesting it is a weaker allele. To test this, we developed an antibody to Akr1B and performed Western blot analyses. We find two alleles of *akr1B* (PL and d0) reduce protein levels by ∼40%, whereas the *akr1B* ^*EY*^ allele only reduces it by ∼9% (Supplementary Figures S4A). For the rest of the study, we focused on the stronger alleles (PL and d0). Because of the weak nature of these alleles, we also assessed one of the stronger alleles over two different deficiencies encompassing *akr1B*; in both cases, this results in delayed migration (Supplementary Figures S4B, MIs = 0.681 and 0.609, *p* < 0.01 and *p* < 0.0001 respectively). We also assessed S10A follicles and find that border cell number and completion of migration are unaffected in an *akr1B* mutant (Supplementary Figures S2C–E). These data indicate that mild reductions in Akr1B level are sufficient to impair border cell migration during S9 that are resolved by S10A.

We next assessed the role of Akr1B in regulating cluster morphology. While the *akr1B* ^*PL*^ allele has no effect on cluster length, the *akr1B* ^*d0*^ allele results in a more compacted cluster (Figure 3F). Similarly, *akr1B* ^*PL*^ over one deficiency, but not the other, results in compacted clusters (Supplementary Figures S4C). Together these findings indicate that Akr1B is required for on-time border cell migration and plays a role cluster morphology.

### Akr1B is required in the border cells for on-time border cell migration and has cell-specific roles in cluster morphology

**FIGURE 4:**
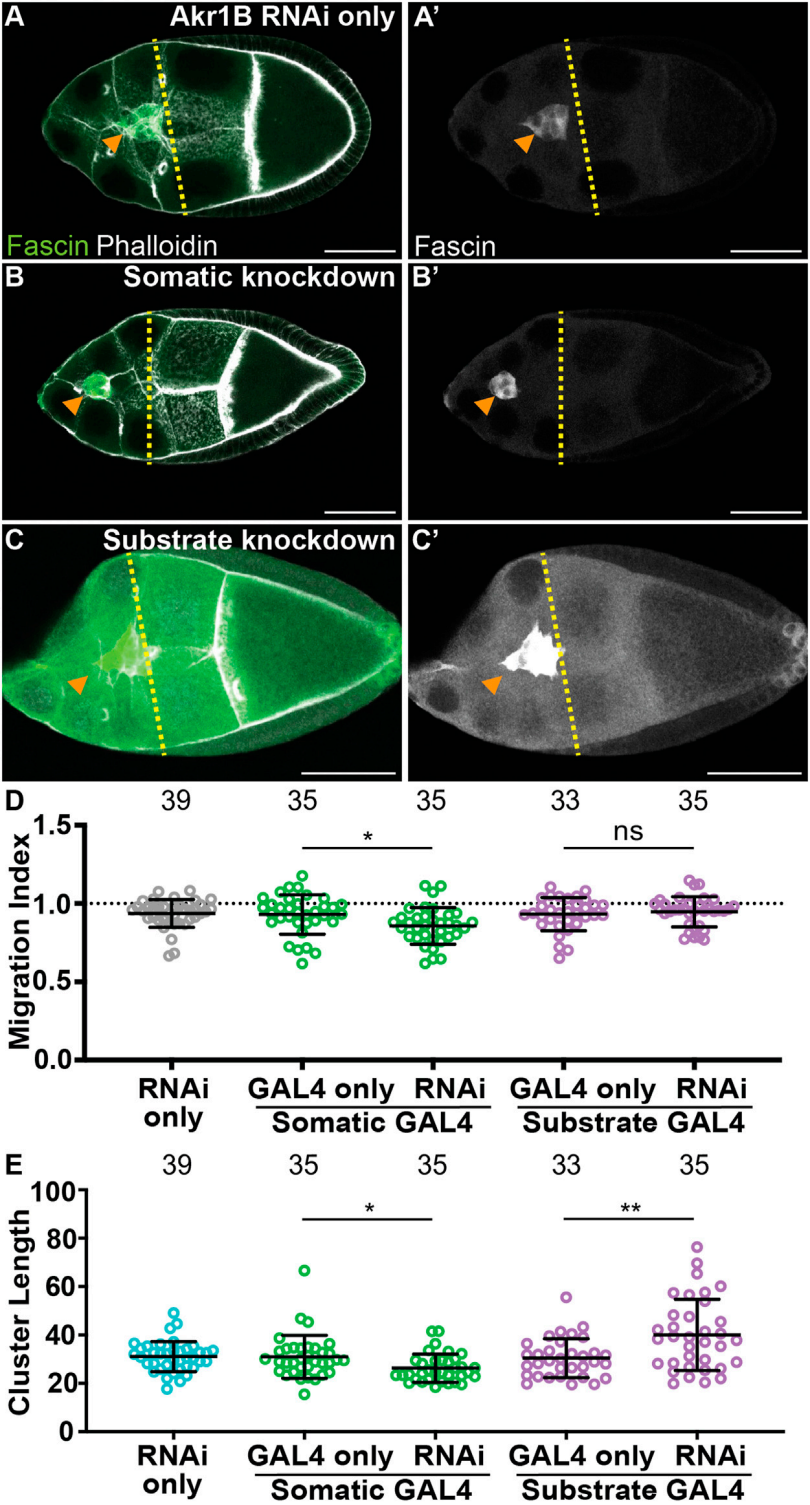
Akr1B is required in the somatic cells for on-time border cell migration, but acts in both the soma and the substrate to regulate cluster morphology. **(A–C’)**. Maximum projections of 3 confocal slices of S9 follicles stained for Fascin (green in merge) and F-actin (phalloidin, white in merge). Orange arrowheads indicate the border cell cluster and yellow dashed lines indicate the position of the outer follicle cells. Images brightened by 30% for merge and 85% for Fascin single-channel images to increase clarity. Scale bars = 50 μm. **(A,A’)**. Akr1B RNAi control (*akr1B RNAi/+*). **(B,B’)**. Somatic knockdown of Akr1B (*c355 GAL4/+; akr1B RNAi/+*). **(C,C’)**. Substrate knockdown of Akr1B (*osk GAL4/akr1B RNAi*). The *akr1B* RNAi line used was HMS05657. **(D–E)**. Graphs of migration index **(D)** and border cell cluster length **(E)** for the indicated genotypes; n = number of follicles. In **(E)**, the dotted line indicates on-time border cell migration. For **(D, E)**, lines = averages and error bars = SD. ns > 0.05, * *p* < 0.05, and ** *p* < 0.01, unpaired *t*-test, two-tailed. Somatic knockdown of Akr1B delays migration **(B-B’, D)** compared to controls **(A-A’,D)**, whereas substrate knockdown exhibits on-time migration **(C-C’,D)**. Akr1B somatic knockdown results in more compact clusters but substrate knockdown results in elongated clusters **(E)**.

To determine where PGF_2α_ synthesis is required for on-time border cell migration, we took two approaches. First, we assessed the localization of Akr1B within S9 follicles. Similar to cPGES, Akr1B is cytoplasmic in all the somatic and germline cells of the follicle (Supplementary Figures S4D). Thus, PGF_2α_ could be produced in either or both cell-types to regulate border cell migration. Next, we used the UAS/GAL4 system to knockdown Akr1B in either all the somatic cells, including the border cells, or the substrate. While the controls (GAL4 and RNAi only) exhibit on-time migration (Figures 4A, D; MIs = 0.930 and 0.936, respectively), RNAi knockdown of Akr1B in the somatic cells slightly delays migration (Figures 4B, D, MI = 0.857, *p* < 0.05 and 0.01, respectively). Knockdown of Akr1B within the substrate results in normal migration compared to the two controls (Figures 4C, D, MI = 0.947). To assess the effectiveness of the RNAi line we expressed the RNAi using a ubiquitous GAL4 driver and performed Western blotting. We find that the RNAi, with this driver, reduces protein levels by ∼50% (Supplementary Figures S5A). These data suggest Akr1B acts in the border cells to promote migration.

We also assessed the cell-specific roles of Akr1B in cluster morphology. Somatic knockdown of Akr1B results in a more compact cluster compared to the controls (Figure 4E, *p* < 0.05 and 0.01, respectively), whereas cluster length is increased in the substrate knockdown (Figure 4E, *p* < 0.01 and 0.001). These findings are similar to what was observed when dCOX1 was knocked down in the different cell populations (Fox et al., 2020), and therefore, suggests that PGF_2α_ is the PG controlling cluster morphology. Our attempt to confirm the migration and cluster morphology results with a second RNAi line were unsuccessful, as we observed with on-time migration and normal cluster morphology in both somatic and substrate knockdowns (Supplementary Figures S5B, C). Supporting this, western blot analysis suggests this RNAi line fails to reduce Akr1B levels (Supplementary Figures S5A).

### Akr1B, but not cPGES, is required for integrin localization to the border cell membranes

**FIGURE 5:**
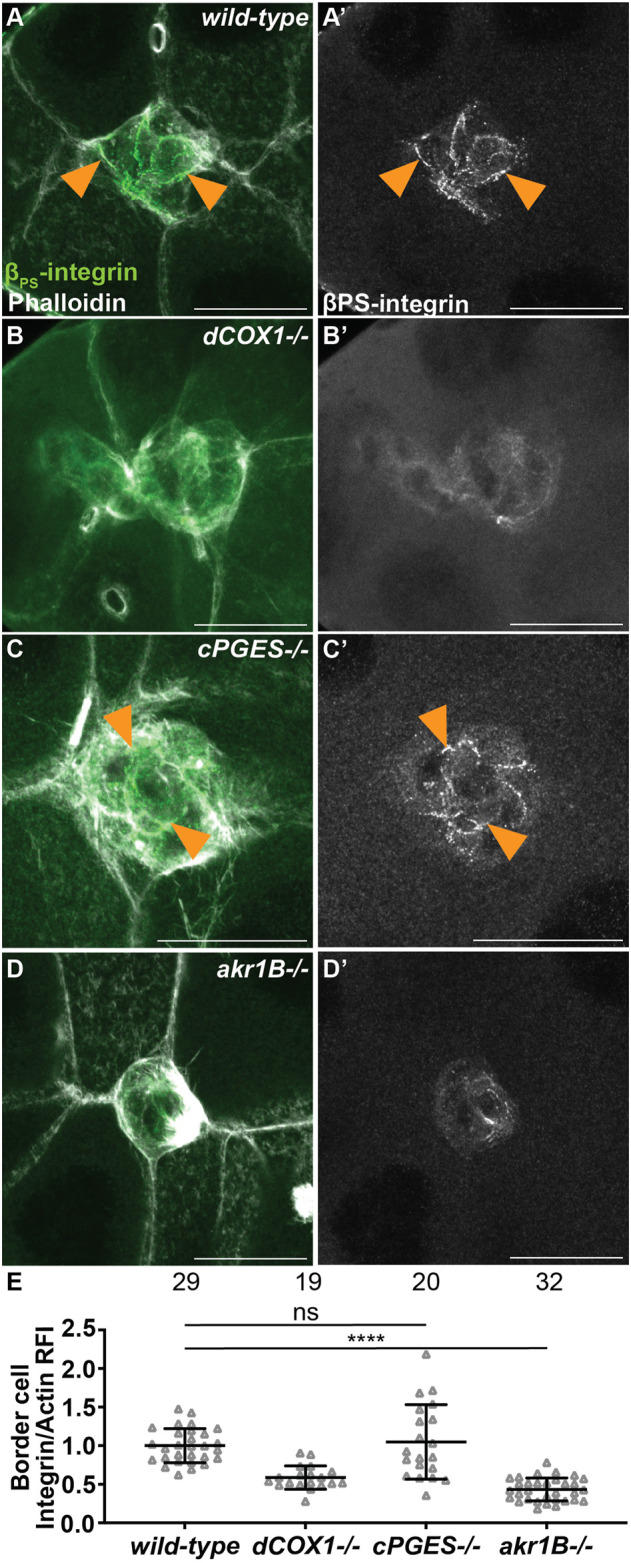
Akr1B is required for integrin localization. **(A–D’)**. Maximum projections of 3 confocal slices of S9 follicles stained with β_PS_-integrin (green in merge) and F-actin (phalloidin, white in merge). Arrowheads indicate examples of β_PS_-integrin membrane localization. Images brightened by 30% to increase clarity. Scale bars = 25 μm. **(A,A’)**. *wild-type* (*yw*). **(B,B’)**. *dCOX1−/−* (*dCOX1* ^*f01000/f01000*^). **(C,C’)**. *cPGES−/−* (*cPGES* ^*EY05607/*EY05607^). **(D,D’)**. *akr1B−/−* (*akr1B* ^*d00405/d00405*^). **(E)**. Graph of the relative fluorescence intensity (RFI) of β_PS_-integrin to F-actin at the border cell membrane for the indicated genotypes (see Materials and Methods for details); *akr1B−/−* = *akr1B* ^*d00405/d00405*^ and *akr1B* ^*PL00034/PL00034*^, and n = number of follicles. Lines = averages and error bars = SD. ns > 0.05, **** *p* < 0.0001, unpaired *t*-test, two-tailed. Like wild-type **(A)**, loss of cPGES **(C-C’,E)** exhibits integrin localization to the border cell membranes. However, *akr1B* mutants **(D-D’,E)** have reduced membrane localization that phenocopies *dCOX1−/−* **(B-B’,E)**.

We next sought to identify the mechanisms whereby PGE_2_ and PGF_2α_ promote on-time border cell migration. We first assessed their role in regulating integrins, as integrins are required for border cell migration and cluster cohesion (Dinkins et al., 2008; Llense and Martin-Blanco, 2008) and PGs are required for integrin localization to the border cell membranes (Fox et al., 2020). In both wild-type and *cPGES* mutant follicles β-integrin (*Drosophila* Myospheroid) localizes to the membranes (Figures 5A, A’, C, C’). Conversely, *akr1B* mutant follicles exhibit a *dCOX1-*like phenotype (Figures 5B, B’), where integrin staining is diffuse throughout the cytoplasm of the cluster (Figures 5D, D’). We quantified integrin localization using our previously described method (Fox et al., 2020); see Materials and Methods for details. Loss of *cPGES* is similar to wild-type *(p*-value = 0.632), but *akr1B* mutant follicles have in an integrin intensity ratio below one (Figure 5E; 0.432, *p* < 0.0001). These results indicate that Akr1B is required for the localization of integrins to the border cell membranes.

### cPGES limits myosin activity within both the substrate and border cells, whereas Akr1B limits it in only the border cells

**FIGURE 6:**
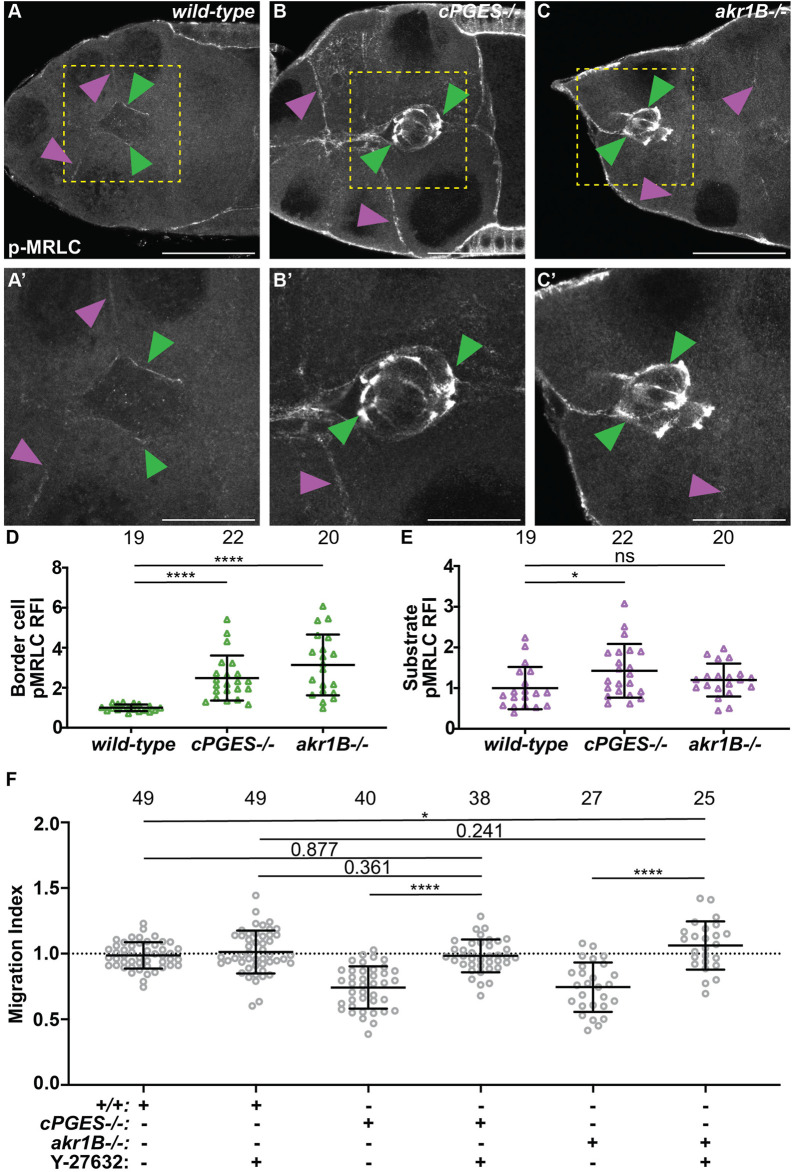
cPGES limits myosin activity within both the border cells and their substrate, whereas Akr1B only limits it within the border cells to promote on-time migration. **(A–C’)**. Maximum projections of 3 confocal slices of S9 follicles stained with pMRLC (white); yellow boxed regions shown at higher resolution in **(A’,B’,C’)**. Arrowheads indicate examples of myosin activity on the border cells (green) and substrate (purple). Scale bars = 25 μm. Images brightened by 30% to increase clarity. **(A,A’)**. *wild-type* (*yw*). **(B,B’)**. *cPGES−/−* (*cPGES* ^*EY05607/EY05607*^). **(C,C’)**. *akr1B−/−* (*akr1B* ^*PL00034/PL00034*^). **(D,E)**. Graphs of relative fluorescence intensity (RFI) of pMRLC on the border cells **(D)** and the substrate **(E)** (see Materials and Methods for details); *akr1B−/−* = *akr1B* ^*d00405/d00405*^ or *akr1B* ^*PL00034/PL00034*^. **(F)**. Graphs of migration index when Y-27632 was used to reduce myosin activity for the indicated genotypes: *+/+* *(yw)*, *cPGES−/−* (*cPGES* ^*EY05607/EY05607*^) and *akr1B−/−* = *akr1B* ^*d00405/d00405*^ or *akr1B* ^*PL00034/PL00034*^ treated with control medium or with 200 μM of Y-27632. In **(D–F)**, triangle or circle = single follicle, n = number of follicles, lines = averages, error bars = SD, and ns > 0.05 or indicated values, * *p* < 0.05, **** *p* < 0.0001, unpaired *t*-test, two-tailed. In **(F)**, the dotted line indicates on-time border cell migration. Loss of cPGES **(B,B’)** increases myosin activity in both the border cells **(D)** and the substrate **(E)** compared to wild-type **(A,A’,D–E)**. Whereas *akr1B* mutants increase myosin activity only in the border cells **(C-C’,D–E)**. Reducing myosin activity by pharmalogic inhibition restores on-time border cell migration in *cPGES* and *akr1B* mutant follicles **(F)**.

The balance of forces between the border cells and their substrate, the nurse cells, is critical for migration, and depends on the level of myosin activity (Majumder et al., 2012; Aranjuez et al., 2016). We previously found that Fascin limits myosin activity within the border cells to control myosin activity in the substrate and thereby, controls substrate stiffness to promote migration (Lamb et al., 2021). As PGs and Fascin act in the same pathway to promote border cell migration (Fox et al., 2020; Lamb et al., 2020), we hypothesize that PG signaling regulates myosin activity. Active myosin is phosphorylated on the myosin regulatory light chain (MRLC) (Vicente-Manzanares et al., 2009; Aguilar-Cuenca et al., 2014). We find that wild-type S9 follicles exhibit a low level of active myosin on the border cell cluster and the substrate (Figures 6A, A’). We quantified pMRLC relative fluorescence intensity using our previously described method (Lamb et al., 2021); see Materials and Methods for details. Loss of cPGES results in a striking increase in active myosin on both the border cells and their substrate (Figures 6B, B’, D, E). Whereas in *akr1B* mutants, myosin activity is only increased on the border cells (Figures 6C–E). These findings, together with our cell-specific knockdown results (Figures 2, 4), lead to the model that cPGES produces PGE_2_ within the substrate to limit myosin activation and therefore, cellular stiffness, of both the substrate and the migratory cells, whereas Akr1B produces PGF_2α_ to limit the stiffness of only the border cells.

To determine if these increases in myosin activity and therefore, cellular stiffness contribute to the migration delays in the *cPGES* and *akr1B* mutants we used a pharmacologic approach to reduce myosin activity. Follicles were incubated in control medium or 200μΜ Y-27632, a Rho inhibitor that reduces myosin activity in *Drosophila* follicles in both the border cells and their substrate (He et al., 2010; Lamb et al., 2021). We then assessed border cell migration. We find that inhibiting myosin activity with Y-27632 restores on-time border cell migration in both the *cPGES* and *akr1B* mutants (Figure 6F). In both mutants and the control follicles treatment with Y-27632 treatment causes mild increases in cluster length (Supplementary Figure S6); these changes are not statistically significant. Together the data support the model that the increased myosin activity observed when either cPGES or Akr1B is reduced contributes to the migration delays observed.

## Discussion

**FIGURE 7:**
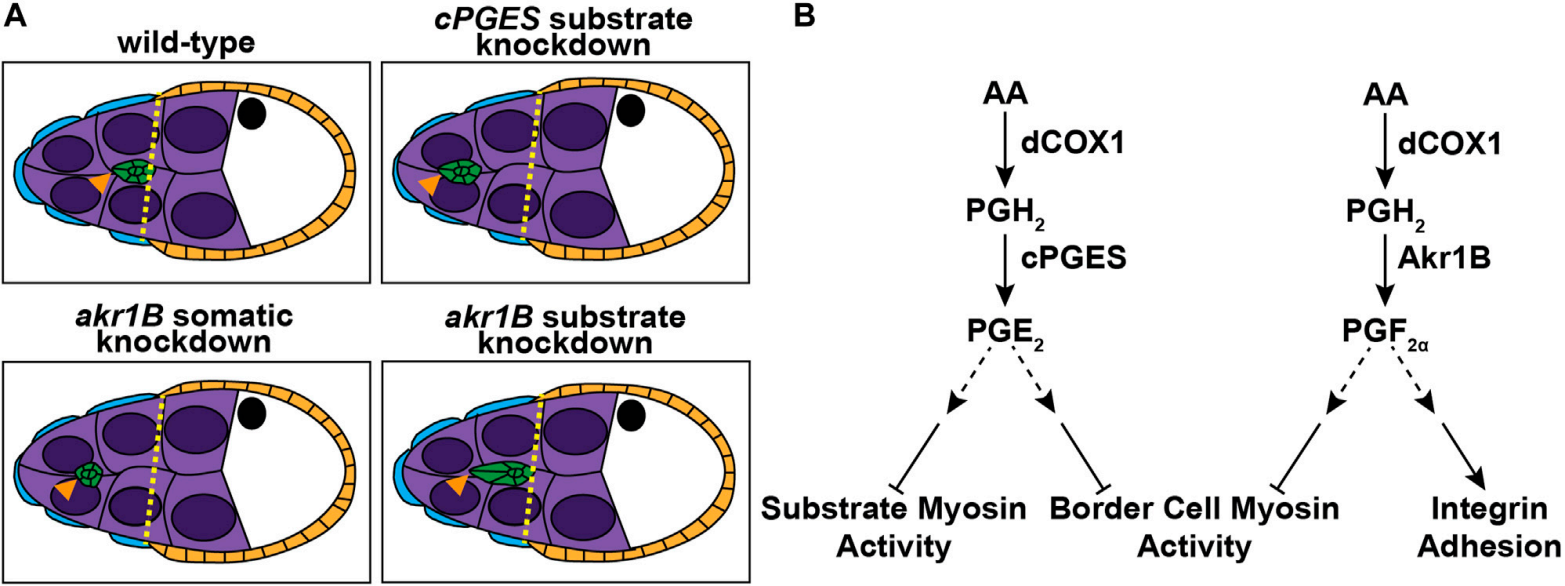
Schematics of the roles of PGE_2_ and PGF_2α_ in border cell migration. Schematics of S9 follicles of the indicated genotypes **(A)** and pathway diagrams **(B)**. In *wild-type* follicles the border cells (green) are in-line with the position of the outer follicle cells (orange and yellow dashed line). Substrate knockdown of *cPGES* and somatic knockdown of *akr1B* delay migration. Thus, cPGES is required in the substrate and Akr1B is required in the somatic cells for on-time migration. Additionally, Akr1B regulates border cell cluster cohesion, as somatic knockdown results in more compact clusters while substrate knockdown results in elongated clusters. Our data supports the model that cPGES in the substrate regulates border cell migration by limiting myosin activity in both the substrate and the border cells, whereas Akr1B only limits myosin activity in the border cells. Akr1B also promotes integrin-based adhesions on the border cells. Which cells produce PGE_2_ and PGF_2α_ to regulate these different downstream effectors remains to be determine.

Using *Drosophila* border cell migration as model, we provide the first evidence that both PGE_2_ and PGF_2α_ synthesis, and therefore signaling, are required for a developmental, collective cell migration. We find that the PGE_2_ synthase cPGES is required in the substrate (the nurse cells) but not the border cells for on-time migration (Figures 1, 2), whereas PGF_2α_ synthesis by Akr1B is required in the border cells (Figures 3, 4). Akr1B acts in both the border cells and the substrate to regulate cluster morphology. Knockdown of Akr1B in the border cells results in compacted clusters, whereas knockdown in the substrate results in cluster elongation (Figure 4). Potentially contributing to these changes in cluster morphology and to its role in migration, Akr1B promotes integrin-based adhesions on the border cells (Figure 5). Another downstream mechanism whereby both PGs promote cell migration is by limiting myosin activity to control cellular stiffness. Specifically, cPGES limits myosin activity in both the border cells and their microenvironment, while Akr1B limits it only within the border cells. Supporting that these changes contribute the delayed migration, pharmacologically reducing myosin activity restores on-time migration in the *cPGES* and *akr1B* mutants (Figure 6). Together these findings reveal that two PGs, PGE_2_ and PGF_2α_, play crucial roles in promoting border cell migration, and that these PGs are produced in distinct locations, the cellular microenvironment and the migrating cells, respectively (Figure 7).

### Mild reductions in PG synthase levels impair migration

Incomplete loss of either cPGES or Akr1B delays border cell migration. Migration is delayed by heterozygosity for *cPGES*, which reduces protein levels by 30% (Supplementary Figures S1A, B), and weak alleles of *Akr1B*, which reduce protein levels by 9%–40% (Figure 3; Supplementary Figures S4A). These findings suggest that mild decreases in PG-type specific synthase levels have striking impacts on PG production and downstream PG signaling. Supporting this idea, siRNA knockdown of cPGES in colon cancer cells retains 36% of the protein level but impairs invasiveness (Cano et al., 2015). Studies in breast cancer cells support that siRNA knockdown of Akr1B1 retains some protein expression but results in an almost complete loss of PGF_2α_ production and impairs migration and invasion (Wu et al., 2017). These data lead us to speculate that heterozygosity for *cPGES* and mild reductions in Akr1B reduce PGE_2_ and PGF_2α_ levels, respectively, below that needed to promote border cell migration. Alternatively, as arachidonic acid release is the rate limiting step in PG production (Funk, 2001; Tootle, 2013), when the level of one synthase is reduced, it may lead to the production of the wrong PG. This wrong PG may dominantly impair migration. Directly testing these ideas will require the development of a method that allows cell-specific levels of individual PGs to be assessed within S9 follicles, as current methods of quantifying PGs–enzyme-linked immunosorbent assay or high-performance liquid chromatography-mass spectrometry–require large amounts of cells and do not provide cellular resolution.

### PGE_2_ produced in the microenvironment promotes cell migration

Numerous studies support that PGE_2_ promotes migration. However, it remains largely unclear which cells produce PGE_2_ *versus* which cells respond to the PGE_2_ signal. Indeed, studies in zebrafish reveal that COX activity and PGE_2_ synthesis/signaling are required for gastrulation (Cha et al., 2005; Cha et al., 2006). However, the cell-specific roles are unknown as the studies employed COX inhibitors and whole organism knockout of COX1 or a PGE_2_ synthase.

Studies measuring PGE_2_ production from tumors and cancer cell lines led to the idea that PGE_2_ is produced and signals autocrinely within cancer cells to drive migration. For example, high COX2 activity is associated with breast cancer metastasis, and breast cancer cell lines with high COX2 levels and PGE_2_ production exhibit increased migration and invasion that are impaired by COX2 inhibition (Singh et al., 2005). Further, studies reveal exogenous PGE_2_ promotes the migration of lung, colon, and prostate cancer cells (Kim et al., 2010; Fujino et al., 2011; Vo et al., 2013).

However, there is growing evidence that PGE_2_ produced in the microenvironment promotes both cancer and immune cell migration (Elwakeel et al., 2019). Colon cancer cells secrete interleukin-1 to activate PGE_2_ production in mesenchymal stem cells (MSCs). This PGE_2_ signals both autocrinely within the MSCs and paracrinely to the cancer cells to drive dedifferentiation and invasion (Li et al., 2012). PGE_2_ also acts paracrinely to promote immune cell migration. Exogenous PGE_2_ is required for dendritic cells to respond to chemokines and chemoattractants (Legler et al., 2006), and reorganizes the actin cytoskeleton to promote migration (Diao et al., 2021). Further, there is evidence that a gradient of PGE_2_ is critical for regulating the state of macrophages, from driving migration at low levels to promoting phagocytosis at high levels (Osma-Garcia et al., 2016).

Given these studies, it is not surprising that we find that PGE_2_ synthesis is required in the microenvironment for border cell migration (Figure 2). Whether all of the nurse cells produce PGE_2_ or produce the same level of PGE_2_ remains unknown, but it is tempting to speculate there may be a gradient from low to high PGE_2_ levels along the anterior to posterior axis that promotes border cell migration.

Our finding that cPGES is required in the microenvironment for on-time border cell migration, contradicts our finding that when all PG synthesis is reduced by RNAi knockdown of dCOX1 in the microenvironment, border cell migration was on-time. This knockdown of dCOX1 was achieved by using an RNAi line that is normally unable to be expressed in the germline, but can be expressed there when combined with reduced Hsp70 (DeLuca and Spradling, 2018). This method likely resulted in a weak knockdown of dCOX1 and we speculate this level of knockdown was not sufficient to limit PG production enough to uncover the microenvironment roles in promoting migration.

We find that cPGES, but not mPGES1 or mPGES2, promotes border cell migration. This finding was unexpected, as mPGES1 is widely implicated in cell migration during both development (Cha et al., 2005; Cha et al., 2006) and cancer (Nakanishi et al., 2008; Kamei et al., 2009; Nakanishi et al., 2011). However, cPGES is also implicated in cancer migration (Cano et al., 2015). One possible reason for our results is that the mPGES1 and mPGES2 alleles tested do not reduce protein levels sufficiently to cause a phenotype; no antibodies are available to assess the alleles. However, we think this is unlikely given that heterozygosity for *cPGES* is sufficient to delay migration (Supplementary Figures S1A). Therefore, we favor the model that cPGES is the primary synthase responsible for producing PGE_2_ within the microenvironment to promote border cell migration.

### PGF_2α_ produced in the migratory cells promotes migration

PGF_2α_ is an understudied PG but has been implicated in acting both autocrinely and paracrinely to promote migration. In breast cancer cell lines, knockdown or inhibition of Akr1B1 decreases, whereas overexpression increases migration and invasion (Wu et al., 2017). In an endometrial cancer cell line, exogenous PGF_2α_ or PGF_2α_ receptor agonist increase migration (Sales et al., 2008). Further, in patients with colon cancer, high expression of Akr1B1 is associated with enhanced motility and poor clinical outcome (Demirkol Canli et al., 2020). These data suggest that PGF_2α_ produced in both the cancer cells and their microenvironment contributes to migration. However, the literature suggests PGF_2α_ acts paracrinely to regulate immune cell migration. PGF_2α_ produced by endothelial cells in the contexts of hypoxia (Arnould et al., 2001) or by endometrial cancer cells promotes neutrophil migration (Wallace et al., 2009). We find that during *Drosophila* border cell migration Akr1B is required within the migratory cells for on-time migration (Figure 4). One caveat to our findings is that the available RNAi lines may not knockdown Akr1B enough. Therefore, Akr1B may act in the microenvironment to regulate border cell migration, but to observe such a role we need a stronger loss of Akr1B than we can currently achieve. However, we think this is unlikely as the *akr1B* ^*EY*^ allele only results in a 9% reduction in protein but delays migration, and the functional RNAi line reduces protein levels by ∼50% (Figure 2; Supplementary Figures S4, 5).

### PGF_2α_ regulates cluster cohesion

In addition to promoting on-time border cell migration, PGs also regulate cluster cohesion. Loss of dCOX1 results in cluster elongation, with clusters sometimes breaking apart (Fox et al., 2020). Similar cluster elongation is seen when dCOX1 is knocked down in the substrate. However, knockdown in the border cells results in cluster compaction, revealing PGs have cell-specific roles in controlling cluster cohesion. Here we find that PGF_2α_ regulates cluster cohesion. Global reduction of the PGF_2α_ synthase Akr1B results in cluster compaction, as does knockdown in the border cells (Figures 3, 4). However, knockdown in the substrate causes cluster elongation (Figure 4). Together these data, along with our findings on dCOX1 (Fox et al., 2020), lead to the model that PGF_2α_ produced within the border cell cluster acts to limit cluster cohesion, whereas PGF_2α_ produced in the substrate promotes cluster cohesion.

While it remains unclear how PGF_2α_ signaling regulates cluster cohesion, our data suggest a few possible mechanisms. First, *akr1B* mutants reduce integrin-based adhesions on the border cells (Figure 5). RNAi knockdown of either subunit of the integrin receptor delays border cell migration, and, when combined with reduced JNK signaling, elongates clusters (Dinkins et al., 2008; Llense and Martin-Blanco, 2008). Therefore, one means by which PGF_2α_ signaling may control cluster cohesion is by tightly regulating integrin-based adhesions. Supporting this idea, in cancer, PGs promote integrin adhesion stability (Mayoral et al., 2005; Bai et al., 2009; Liu et al., 2010). Second, these morphology changes may be due to PGF_2α_ signaling controlling actin cytoskeletal remodeling within the border cells. Indeed, during later stages of *Drosophila* oogenesis PGF_2α_ maintains cortical actin integrity and promotes actin bundle formation (Tootle and Spradling, 2008; Groen et al., 2012; Spracklen et al., 2014). Third, either by regulating the actin cytoskeleton or by other means, PGF_2α_ may control cluster cohesion by modulating cellular stiffness. We find *akr1B* mutants exhibit increased border cell stiffness, as seen by increased myosin activity (Figure 6). The balance of forces between the border cells and their substrate must be tightly regulated for normal cluster morphology, as misbalanced forces cause cluster elongation (Majumder et al., 2012; Cai et al., 2014; Aranjuez et al., 2016). Finally, PGF_2α_ regulation of both cellular stiffness and actin cytoskeletal dynamics may modulate integrin-based adhesions. Thus, all three mechanisms may contribute to PGF_2α_ control of cluster cohesion.

### PGs regulate the balance of forces to promote migration

Cell migration depends on both the stiffness of the migratory cells and their microenvironment, and the balance of those forces (Kai et al., 2016). Numerous studies have shown that substrate stiffness regulates migratory cell stiffness and ability to migrate (Aguilar-Cuenca et al., 2014; Barriga et al., 2018); this is particularly evident in cancer migration and metastasis (Gasparski et al., 2017; Oakes, 2018; Eble and Niland, 2019; Ren et al., 2021). Evidence is also emerging that migrating cells influence their microenvironment. For example, migrating cells degrade extracellular matrix (ECM) to promote migration (Wolf et al., 2007); this likely decreases microenvironment stiffness. Migrating cells can also increase the stiffness of the microenvironment, by pulling on and aligning ECM fibers (Hall et al., 2016; van Helvert and Friedl, 2016). Further, cancer cells induce changes in the stroma, including increasing fibrosis and, thereby, stiffening the tissue (van Helvert et al., 2018; Chandler et al., 2019; Piersma et al., 2020). Ultimately, this increase in the stiffness of the microenvironment promotes cell migration, increasing the force generation in the migratory cells by a process termed mechanoreciprocity (Cox and Erler, 2014; van Helvert et al., 2018). Such a coordinated and interdependent balance of forces is seen between the border cells and their microenvironment, the nurse cells (Majumder et al., 2012; Aranjuez et al., 2016; Lamb et al., 2021).

A key regulator of cellular stiffness is myosin, a force generating actin motor (Vicente-Manzanares et al., 2009; Aguilar-Cuenca et al., 2014). Indeed, myosin controls the stiffness of both migrating cells and their cellular substrates (Lo et al., 2000; Vicente-Manzanares et al., 2009; Mohan et al., 2015). Further, myosin serves as a force sensor, driving the cellular response to applied forces (Butcher et al., 2009; Vicente-Manzanares et al., 2009; Aguilar-Cuenca et al., 2014). Myosin plays these important roles during border cell migration (Majumder et al., 2012; Aranjuez et al., 2016; Lamb et al., 2021). When myosin activity is severely increased in the nurse cells, the microenvironment, it increases active myosin in the border cells and delays migration (Aranjuez et al., 2016). Increasing myosin activity on the border cells also drives myosin activation and stiffening of the nurse cells. This migratory cell influence on the microenvironment depends on Fascin (Lamb et al., 2021). This function of Fascin is likely regulated by PG signaling, as Fascin is a downstream effector of PGs during border cell migration (Fox et al., 2020).

We find that PGE_2_ and PGF_2α_ synthesis have cell-specific roles in regulating myosin activity (Figure 6). Loss of cPGES results in increased myosin activation on both the border cells and the nurse cells, whereas reduction in Akr1B only increases it on the border cells. This loss of mechanoreciprocity could be due to insufficient reduction in Akr1B and thereby, PGF_2α_ levels. Alternatively, it could indicate cell-specific roles of the different PGs. Taking our cell-specific knockdown findings into account (Figures 2, 4), we speculate: Akr1B-dependent PGF_2α_ production within the border cells limits myosin activity and border cell stiffness. PGF_2α_ synthesis may also be required for the nurse cells to appropriately respond to the forces placed on them. PGE_2_ synthesis by cPGES in the nurse cells signals to the border cells to modulate border cell stiffness, which in turn controls nurse cell stiffness. We hypothesize that both PGE_2_-and PGF_2α_-dependent regulation of myosin activity occurs, at least in part, via modulating Fascin activity. The increased myosin activity within both the *cPGES* and the *akr1B* mutants contributes to the migration delays, as pharmacological inhibition of myosin activation restores on-time migration in both mutants.

The role of PGs in regulating myosin activity is likely conserved. In colonic lamina propria fibroblasts, PGE_2_ signaling is required for reducing myosin activity to allow cell polarization and migration during wound healing (Rieder et al., 2010). PGE_2_ regulates myosin activation in dendritic cells, controlling their maturation (van Helden et al., 2008). PGF_2α_ promotes myosin activation in muscle cells, driving their contraction (Ansari et al., 2004; Xu et al., 2015); how it influences myosin activity in other cells remains unknown. Future studies on *in vivo* migrating cells, like the border cells, are needed to uncover the roles of distinct PGs in modulating myosin activity and cellular stiffness to promote migration.

### Do PGE_2_ and PGF_2α_ signal at different times during border cell migration?

Our data show that both PGE_2_ and PGF_2α_ synthesis are required for on-time border cell migration. However, it remains unknown whether they signal simultaneously or at distinct times, and whether one PG induces the production of the other. Supporting the latter possibilities, in colorectal tumor cells PGF_2α_ signaling induces the production of PGE_2_ (Stamatakis et al., 2015). If this occurs during border cell migration, it could help explain why small reductions in Akr1B levels result in such striking delays in border cell migration (Figure 3; Supplementary Figure S4). However, if this were the only mechanism controlling PGE_2_ production, one would predict loss of Akr1B would phenocopy loss of cPGES and exhibit increased myosin activity in both the border cells and nurse cells (Figure 6). It is also possible that force transmission from the border cells to the nurse cells, which likely occurs by both PGF_2α_-dependent and independent mechanisms, activates PGE_2_ production. Indeed, cytoplasmic phospholipase A2 (cPLA2) is activated by mechanical signaling, resulting in the release of arachidonic acid, the substrate for all PG production (Enyedi et al., 2016; Lomakin et al., 2020); substrate release is the rate limiting step in PG synthesis (Funk, 2001; Tootle, 2013). Future studies, in conjunction with developing methods for visualizing the timing of PG synthesis and signaling, are needed to determine the interplay between PGE_2_ and PGF_2α_ in border cell migration.

## Conclusion

The field’s understanding of cell-specific roles of individual PGs in cell migration has been limited by the widespread use of ubiquitously perturbing PG synthesis and signaling components, and by studying cellular responses to exogenously supplied PGs. *Drosophila* border cell migration provides an *in vivo*, physiological system to decipher the cell-specific roles of different PGs in promoting collective cell migration, allowing the separation of roles within the migratory cells *versus* their microenvironment (Figure 7). Here we find that cPGES-dependent production of PGE_2_ is not required in the migratory cells, but is necessary in microenvironment to promote border cell migration. Further, on-time migration requires PGF_2α_, an understudied PG, to be produced by Akr1B in the border cells. These findings call for a reassessment of the cellular sites of PGE_2_ synthesis, and for widespread examination of the roles of PGF_2α_ in cell migration, from development to cancer metastasis. Our work suggests both PGs promote migration by controlling myosin activity and cellular stiffness, but whether they do so by the same or different mechanisms remains unknown. Further, we find PGF_2α_, but not PGE_2_ synthesis, is required for integrin-based adhesions. It will be important to determine whether these downstream mechanisms of PGE_2_ and PGF_2α_ signaling are conserved across some or all collective cell migrations.

## Data Availability

The original contributions presented in the study are included in the article/Supplementary Material, further inquiries can be directed to the corresponding author.
