# Supplementary material for: Specific prostaglandins are produced in the migratory cells and the surrounding substrate to promote Drosophila border cell migration
Source: Front Cell Dev Biol. 2024 Jan 12;11:1257751. doi: 10.3389/fcell.2023.1257751 (PMC10811798; doi:10.3389/fcell.2023.1257751)
Supplement: Supplementary file 3 [file DataSheet1.pdf]

Supplemental Figures

Supplemental Figure 1: Heterozygosity for *cPGES* delays border cell migration and cPGES is ubiquitously expressed at S9. A. Graph of migration index for the indicated genotypes. Each circle represents a single follicle; n = number of follicles. The dotted line indicates on-time border cell migration, lines = averages, error bars = SD., and * $p<0.05$ and ** $p<0.01$, unpaired t-test, two-tailed. B. Graph quantifying cPGES protein levels by western blot analyses normalized to wild-type levels; loading was normalized based on α-Tubulin levels. C-D'. Maximum projections of 3 confocal slices of S9 follicles stained for cPGES (green) and F-actin (phalloidin, white) for the indicated genotypes; higher resolution images of the border cells (orange dashed boxes in C and D) shown in C' and D'. Images brightened by 30% to increase clarity. Scale bars = 50μm for C, D and 25μm C', D'. Genotypes used: *wild-type* (*yw*), *cPGES-/+* (*cPGES*^{EY05607}/+), and *cPGES-/-* (*cPGES*$^{EY05607/EY05607}$). Heterozygosity for the mutation in *cPGES* delays border cell migration to a similar extent as the homozygotes (A). *cPGES-/+* causes a 30% reduction in protein level, while *cPGES-/-* results in a 77% reduction. cPGES is expressed in all the cells of wild-type S9 follicle, including the nurse cell substrate and the border cells (C-C'), and cPGES staining is strongly reduced in the *cPGES* mutant (D-D').

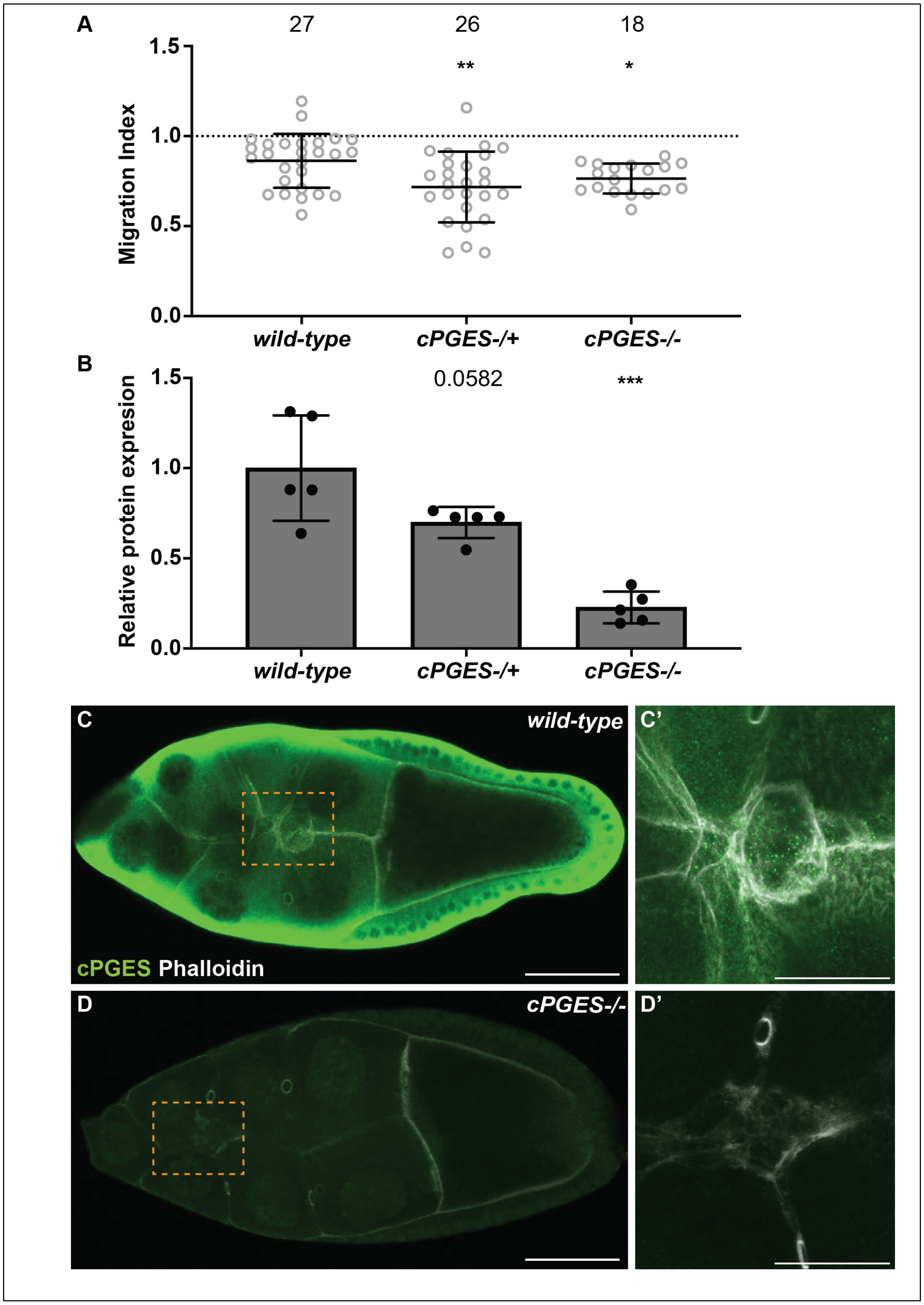
A
27
26
18
**
*
Migration Index
1.5
1.0
0.5
0.0
wild-type
cPGES-/+
cPGES-/-
B
0.0582
***
Relative protein expresion
1.5
1.0
0.5
0.0
wild-type
cPGES-/+
cPGES-/-
C
wild-type
C'
cPGES Phalloidin
D
cPGES-/-
D'

Supplemental Figure 2: Border cell migration is completed by S10 in *cPGES* and *akr1B* mutants. A-C. Maximum projections of 3 confocal slices of S10A follicles stained for Fascin (green in merge) and F-actin (phalloidin, white in merge). Orange arrowheads indicate the border cell cluster. Images brightened by 20% to increase clarity. Scale bars = 50μm. A. *wild-type* (*yw*). B. *cPGES-/-* ($cPGES^{EY05607/EY05607}$). C. *akr1B-/-* ($akr1B^{d00405/d00405}$). D-E. Graphs of number of border cells in the cluster (D) and the number of border cells left along the migration path (E) at S10A for indicated genotypes; circle = single follicle and n = number of follicles. For D, lines = averages and error bars = SD. Like in wild-type (A), in *cPGES* (B) and *akr1B* (C) mutant S10A follicles the border cell clusters complete migration and reach the oocyte; the clusters also contain the same number of border cells (D). In wild-type and *akr1B* mutant S10A follicles there are no somatic cells along the migration pathway, whereas in many *cPGES* mutant S10A follicles there are 1 to 3 somatic cells of unclear origin along the migration path (E).

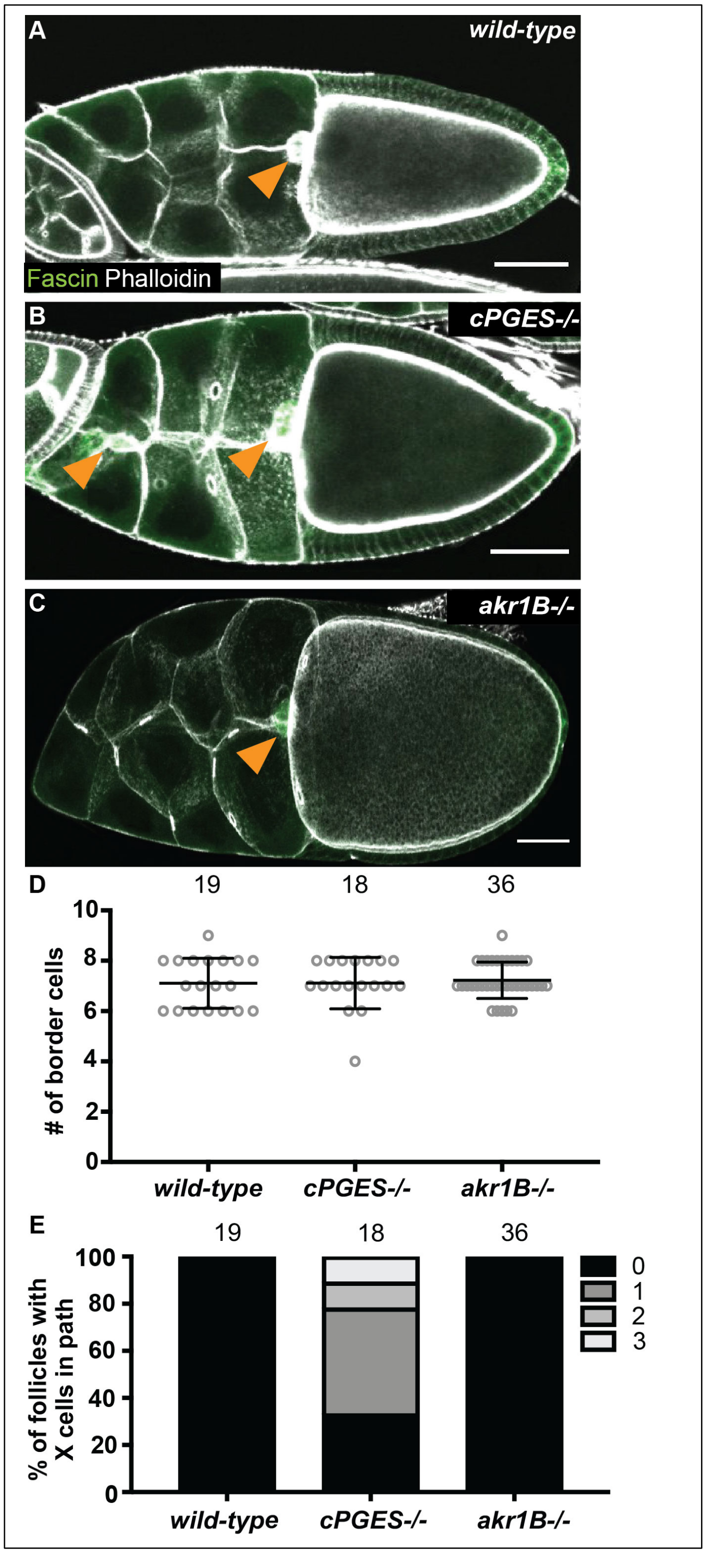
A
wild-type
Fascin Phalloidin
B
cPGES-/-
C
akr1B-/-
D
19
18
36
# of border cells
wild-type
cPGES-/-
akr1B-/-
E
19
18
36
% of follicles with X cells in path
0
1
2
3
wild-type
cPGES-/-
akr1B-/-

Supplemental Figure 3: RNAi knockdown of cPGES. A-C. Maximum projections of 3 confocal slices of S9 follicles stained for cPGES (green in merge) and F-actin (phalloidin, white in merge) for the following genotypes: A-A'. cPGES RNAi control (*cPGES RNAi/+*). B-B'. Somatic knockdown of cPGES (*c355 GAL4/+; cPGES RNAi/+*). C-C'. Substrate knockdown of cPGES (*osk GAL4/cPGES RNAi*). cPGES RNAi used in A-C was HMJ24151. Orange arrowheads indicate the border cell cluster, blue arrowheads indicate the outer follicle cells, and yellow dashed lines indicate the position of the outer follicle cells. Images brightened by 30% to increase clarity. Scale bars = 50μm D-E. Graphs of migration index (D) and border cell cluster length (E) for the indicated genotypes. Genotypes include: cPGES RNAi-2 control (*cPGES RNAi-2/+*); somatic GAL4 only (*c355 GAL4/+*); somatic knockdown of cPGES (*c355 GAL4/+; cPGES RNAi-2/+* or *c355 GAL4/+; UAS dicer/+;cPGES RNAi-2/+*), the results were not different with or without UAS Dicer; substrate GAL4 only (*osk GAL4/+*) substrate knockdown of cPGES (*osk GAL4/+;cPGES RNAi-2/+*). cPGES RNAi-2 was GL01292. Each circle represents a single follicle; n = number of follicles. In D, the dotted line indicates on-time border cell migration. For D-E, lines = averages and error bars = SD. ns>0.05, **** $p<0.0001$, unpaired t-test, two-tailed. cPGES is expressed in all cells of the S9 follicle, including the border cells (A). Whereas, cPGES is expressed only in the substrate in the somatic knockdown (B), and in the somatic cells in the substrate knockdown (C). The second cPGES RNAi line recapitulates what was observed in Fig. 2, as somatic knockdown of cPGES with RNAi-2 exhibits on-time border cell migration, whereas substrate knockdown delays migration (D). Border cell cluster length is unaffected by knockdown of cPGES with RNAi-2 in either the somatic cells or the substrate (E).

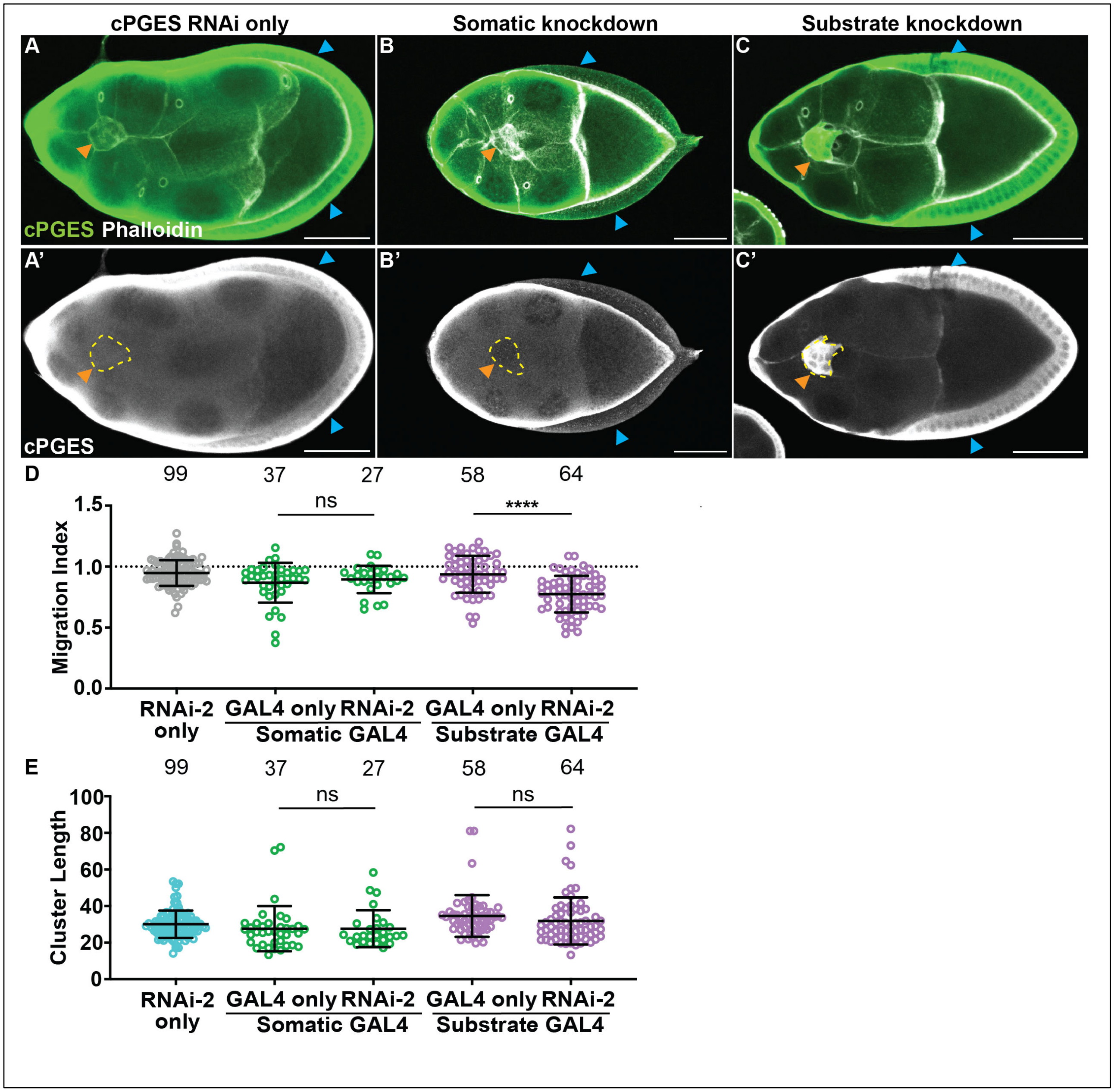

cPGES RNAi only
Somatic knockdown
Substrate knockdown
A
B
C
cPGES Phalloidin
A'
B'
C'
cPGES
D
99
37
27
58
64
ns
****
Migration Index
1.5
1.0
0.5
0.0
RNAi-2 only
GAL4 only
RNAi-2
GAL4 only
RNAi-2
Somatic GAL4
Substrate GAL4
E
99
37
27
58
64
ns
ns
Cluster Length
100
80
60
40
20
0
RNAi-2 only
GAL4 only
RNAi-2
GAL4 only
RNAi-2
Somatic GAL4
Substrate GAL4

Supplemental Figure 4: Mild reductions in Ark1B levels delay border cell migration and cause cluster compaction. A. Graph quantifying protein levels by western blot analyses for Akr1B relative to wild-type values; the α-Tubulin loading control was used to normalize protein levels. Genotypes: *wild-type* (*yw*), *akr1B*$^{PL00034/PL00034}$, *akr1B*$^{d00405/d00405}$, and *akr1B*$^{EY07011/EY07011}$. B-C. Graphs of migration index (B) and border cell cluster length (C) for the indicated genotypes: *akr1B*$^{PL00034/+}$ (*akr1B*^{PL}); *Df(3L)BSC577/+* (Deficiency 1), *Df(3L)ED4475/+* (Deficiency 2), *Df(3L)BSC577/akr1B*^{PL00034}, and *Df(3L)ED4475/akr1B*^{PL00034}. Each circle represents a single follicle; n = number of follicles. In B, the dotted line indicates on-time border cell migration. For B-C, lines = averages and error bars = SD. ns>0.05, ** p<0.01, and **** p<0.0001, unpaired t-test, two-tailed. D-E. Maximum projections of 3 confocal slices of S9 follicles stained for Akr1B (green) and F-actin (phalloidin, white) for the indicated genotypes; *wild-type* (*yw*) and *akr1B*$^{d00405/d00405}$(*akr1B*$^{d0/d0}$). Orange arrowheads indicate the border cell cluster. Images brightened by 30% to increase clarity. Scale bars = 50µm. The *akr1B* alleles are weak loss of functions alleles, as the *akr1B*$^{EY07011/EY07011}$ mutants only exhibit a 9% reduction in protein, whereas *akr1B*$^{PL00034/PL00034}$ and *akr1B*$^{d00405/d00405}$ reduce Akr1B levels by 33% and 40% compared to *wild-type* (A). To further reduce Akr1B we assessed transheterozygotes of *akr1B*^{PL00034} with two different Deficiency lines removing the *akr1B* gene. Like the stronger a*kr1b* mutants, both transheterozygous combinations exhibit delayed border cell migration (B); however, only the transheterozygotes with Deficiency 1 had compacted clusters. Immunofluorescence staining reveals Akr1B is expressed in all the cells of wild-type S9 follicle (D). In *akr1B*$^{d0/d0}$ mutant follicles the Akr1B staining is mildly reduced (E), which is consistent with the western blot analyses (A).

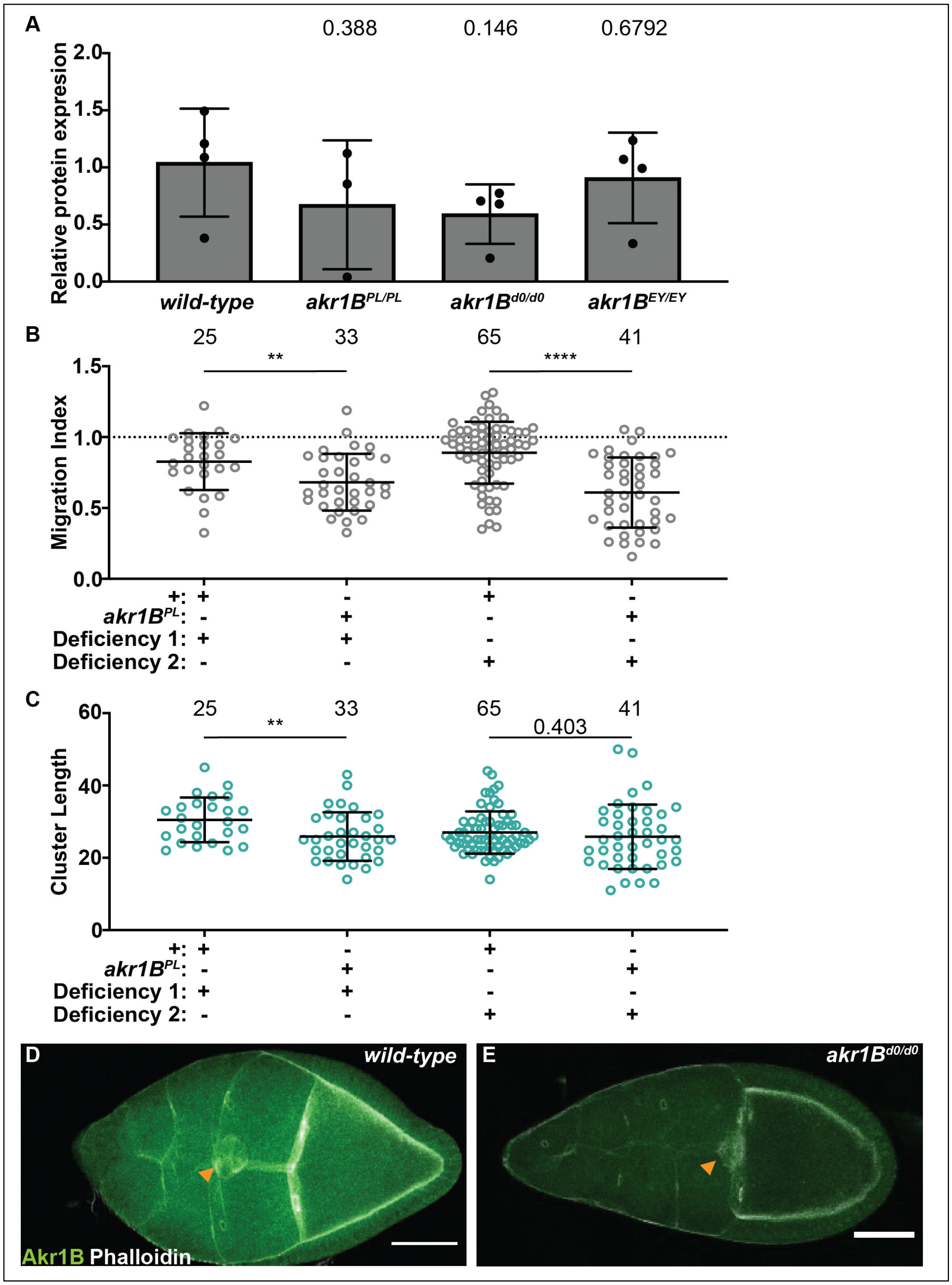

A
0.388
0.146
0.6792
Relative protein expresion
2.0
1.5
1.0
0.5
0.0
wild-type
akr1B PL/PL
akr1B d0/d0
akr1B EY/EY
B
25
33
65
41
**
****
Migration Index
1.5
1.0
0.5
0.0
+: + - + -
akr1B PL: - + - +
Deficiency 1: + + - -
Deficiency 2: - - + +
C
25
33
65
41
**
0.403
Cluster Length
60
40
20
0
+: + - + -
akr1B PL: - + - +
Deficiency 1: + + - -
Deficiency 2: - - + +
D
wild-type
Akr1B Phalloidin
E
akr1B d0/d0

Supplemental Figure 5: Second Akr1B RNAi line does not affect border cell migration or cluster morphology. A. Graph quantifying protein levels by western blot analyses for Akr1B relative to RNAi-1 only control value; the α-Tubulin loading control was used to normalize protein levels. Genotypes: RNAi-1 only (*akr1B RNAi-1/+*); RNAi-1/Actin Gal4 (*akr1B RNAi-1/actin GAL4*); RNAi-1 only (*akr1B RNAi-1/+*); RNAi-2/Actin Gal4 (*akr1B RNAi-2/actin GAL4*). The *akr1B* RNAi-1 is HMS05657 and RNAi-2 is HMC05226. ns $p>0.05$ (p value provided, ****$p<0.0001$, unpaired t-test, two-tailed. B-C. Graphs of migration index (A) and border cell cluster length (B) for the following genotypes include: Akr1B RNAi-2 only control (*akr1B RNAi-2/+*), somatic GAL4 only control (*c355 GAL4/+*), somatic knockdown of Akr1B (*c355 GAL4/+; akr1B RNAi-2/+*), substrate GAL4 only control (*osk GAL4/+*), and substrate knockdown of Akr1B (*osk GAL4/akr1B RNAi-2*). Each circle represents a single follicle; n = number of follicles. In A, the dotted line indicates on-time border cell migration. For A-B, lines = averages and error bars = SD. p values shown, unpaired t-test, two-tailed. Like the controls, both substrate and somatic knockdown of Akr1B with RNAi-2 exhibits on-time migration (A) and normal cluster length (B).

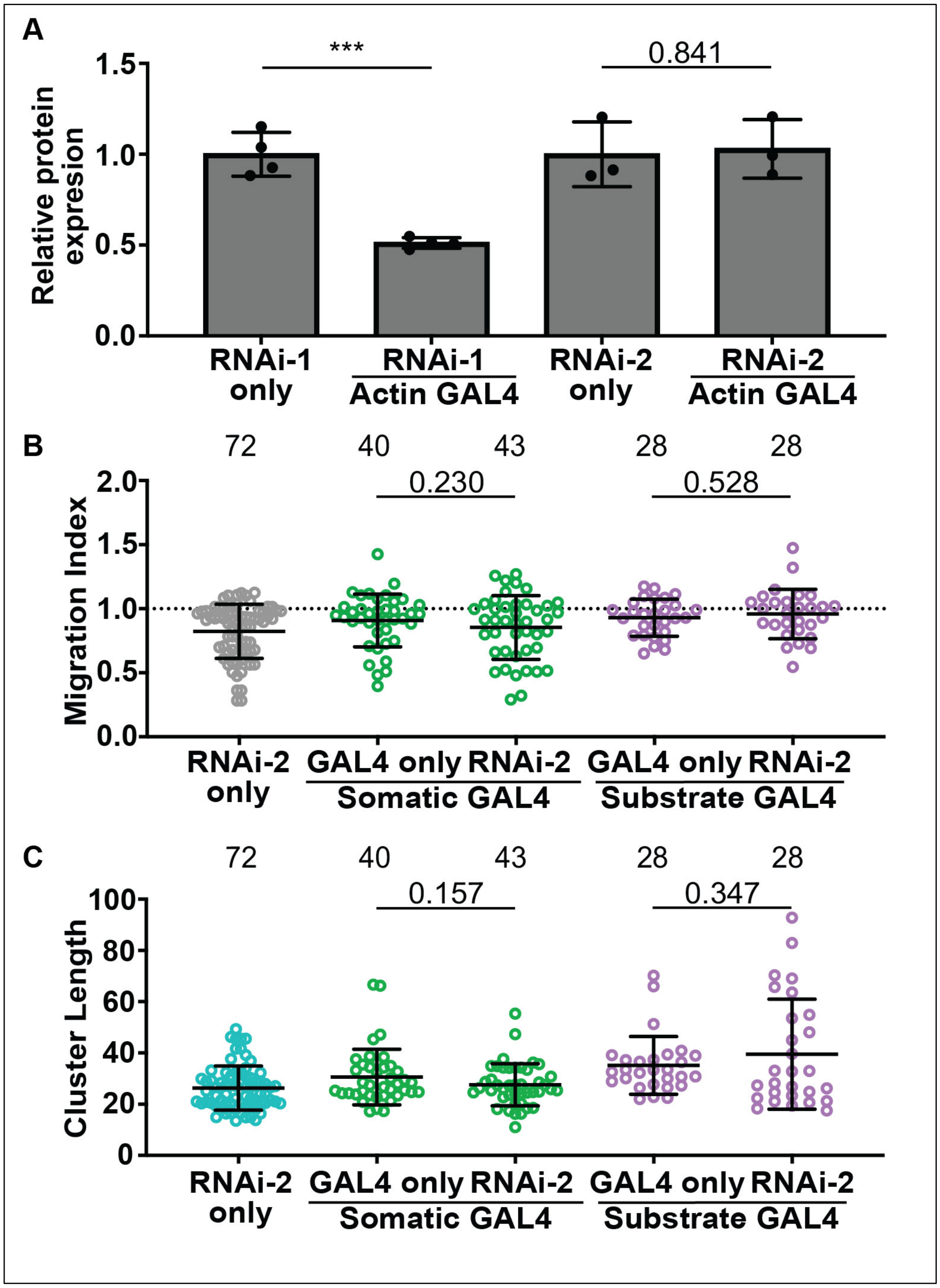
A
***
0.841
Relative protein expresion
1.5
1.0
0.5
0.0
RNAi-1 only
RNAi-1
Actin GAL4
RNAi-2 only
RNAi-2
Actin GAL4
B
72
40
43
28
28
0.230
0.528
Migration Index
2.0
1.5
1.0
0.5
0.0
RNAi-2 only
GAL4 only
RNAi-2
Somatic GAL4
GAL4 only
RNAi-2
Substrate GAL4
C
72
40
43
28
28
0.157
0.347
Cluster Length
100
80
60
40
20
0
RNAi-2 only
GAL4 only
RNAi-2
Somatic GAL4
GAL4 only
RNAi-2
Substrate GAL4

Supplemental Figure 6: Graph of border cell cluster length when Y-27632 was used to reduce myosin activity for the indicated genotypes: wild-type (+/+), *cPGES-/-* (*cPGES*$^{EY05607/EY05607}$) and *akr1B-/-* = *akr1B*$^{d00405/d00405}$ or *akr1B*$^{PL00034/PL00034}$ treated with control medium or with 200μM of Y-27632. Circle = single follicle, n = number of follicles, lines = averages, error bars = SD, and ns>0.05 or indicated values, * p<0.05 and ** p<0.01, unpaired t-test, two-tailed. Impairing myosin activity increases cluster length for all genotypes, but the changes are not significant compared to the control.

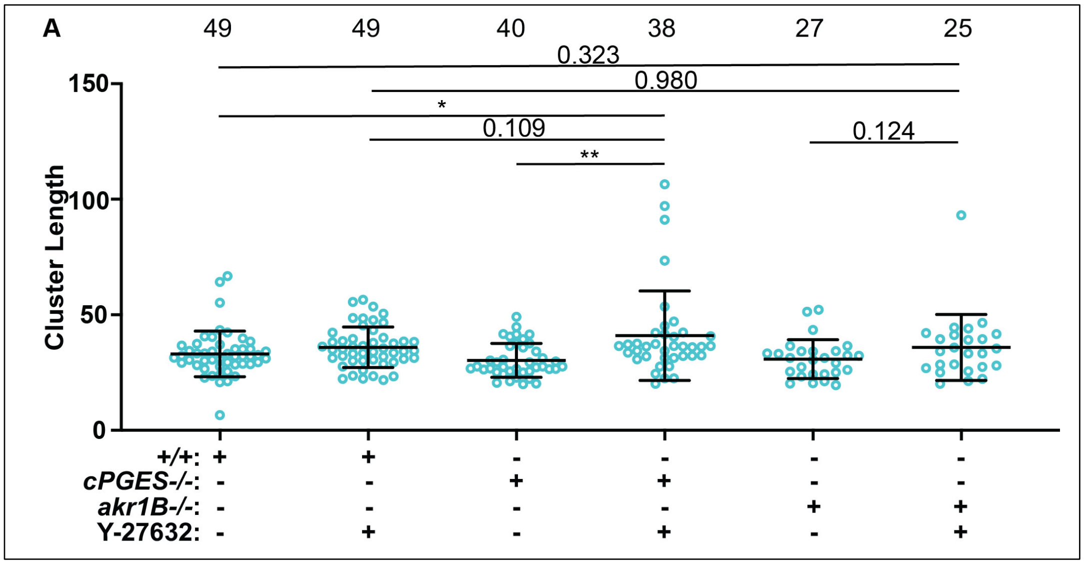

Supplemental Figure 7: Western blot images. A-F. Images of full western blots used for analyses in SFig. 1, SFig. 4 and SFig. 5. Note, lanes that are not used for analysis are labeled with a *. A-B. Western blot for cPGES and α-Tubulin (loading control) for the following genotypes: *wild-type* (*yw*)*, cPGES-/+* (*cPGES*^{EY05607}/+)*, and cPGES-/-* (*cPGES*$^{EY05607/EY05607}$)*. cPGES-/+* was either over a *wild-type* or a balancer chromosome. Blot was cut horizontally at ~35kDa to blot for both proteins at the same time. C-D. Western blots for Akr1B and α-Tubulin (loading control) for the following genotypes: *wild-type* (*yw*)*, akr1B*$^{PL00034/PL00034}$*, akr1B*$^{d00405/d00405}$*, akr1B*$^{EY07011/EY07011}$. In C, the blot was cut horizontally at ~45kDa to blot for both proteins at the same time. In D, the blot was used to detect Akr1B first, then stripped and reprobed to detect α-Tubulin; two different exposures are shown to have the correct exposures for the individual experimental set. E-F. Western blots for Akr1B and α-Tubulin (loading control) for the following genotypes: *actin-5c GAL4/*+, *Akr1B RNAi-1/*+, *Akr1B RNAi-1/actin-5c GAL4*, *Akr1B RNAi-2/*+, *Akr1B RNAi-2/actin-5c GAL4.* RNAi-1 is HMS05657 and RNAi-2 is HMC05226. Blots were cut horizontally at ~45kDa to blot for both proteins at the same time.

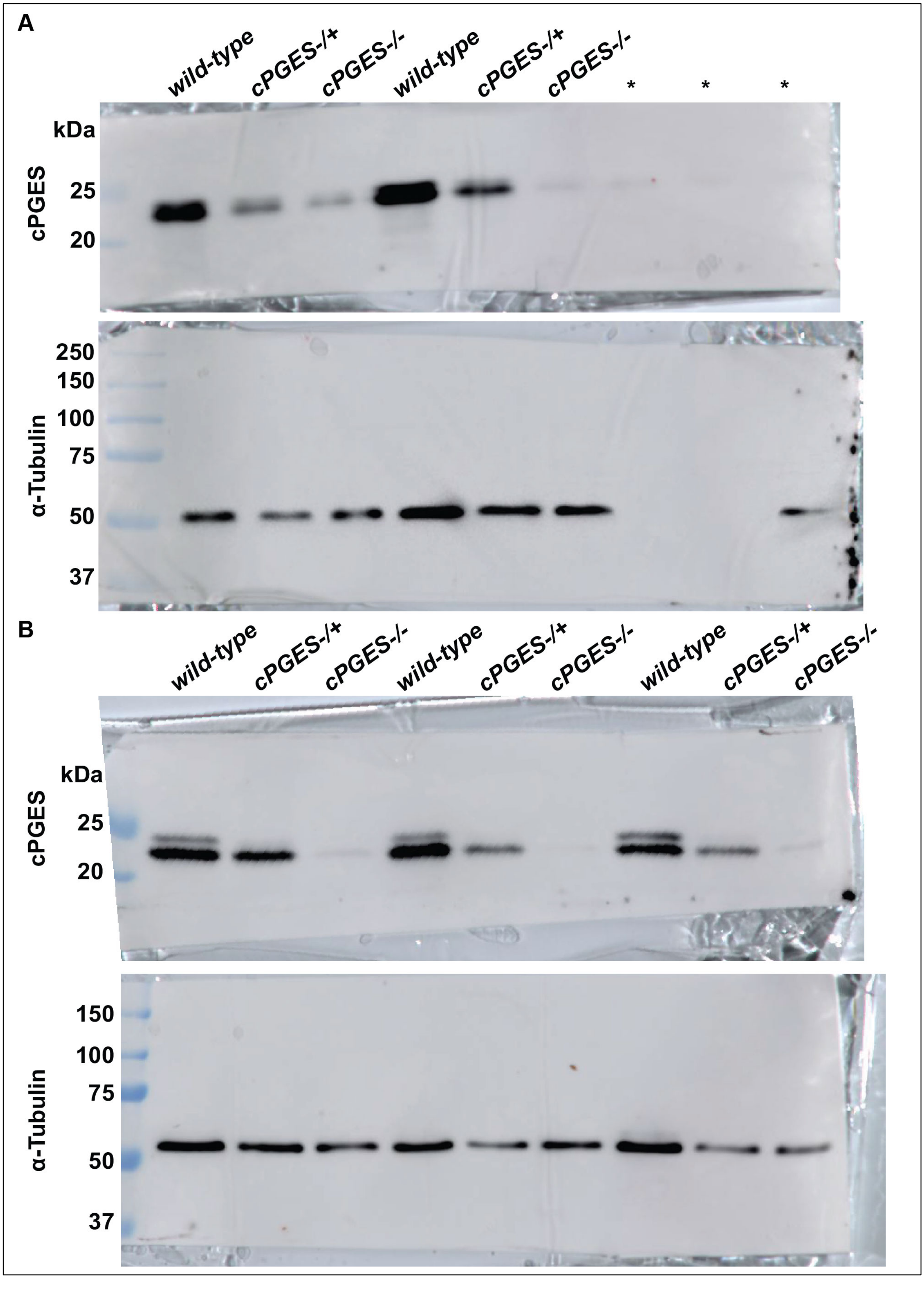
A
wild-type
cPGES-/+
cPGES-/-
wild-type
cPGES-/+
cPGES-/-
*
*
*
kDa
cPGES
25
20
α-Tubulin
250
150
100
75
50
37
B
wild-type
cPGES-/+
cPGES-/-
wild-type
cPGES-/+
cPGES-/-
wild-type
cPGES-/+
cPGES-/-
kDa
cPGES
25
20
α-Tubulin
150
100
75
50
37

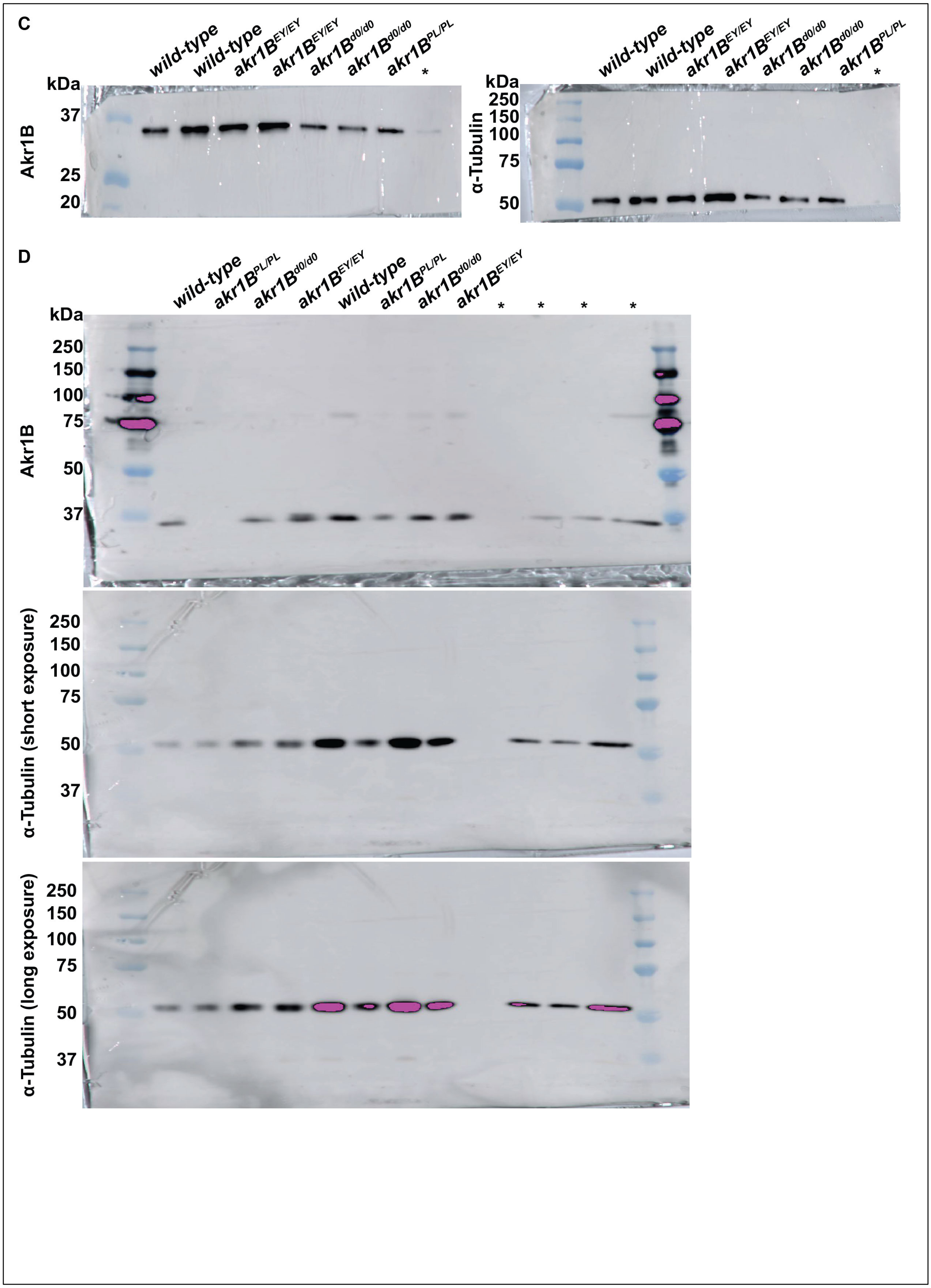
C
wild-type
wild-type
akr1B^EY/EY
akr1B^EY/EY
akr1B^d0/d0
akr1B^d0/d0
akr1B^PL/PL
*
kDa
37
25
20
Akr1B
α-Tubulin
250
150
100
75
50
D
wild-type
akr1B^PL/PL
akr1B^d0/d0
akr1B^EY/EY
wild-type
akr1B^PL/PL
akr1B^d0/d0
akr1B^EY/EY
* * * *
kDa
250
150
100
75
50
37
Akr1B
α-Tubulin (short exposure)
α-Tubulin (long exposure)

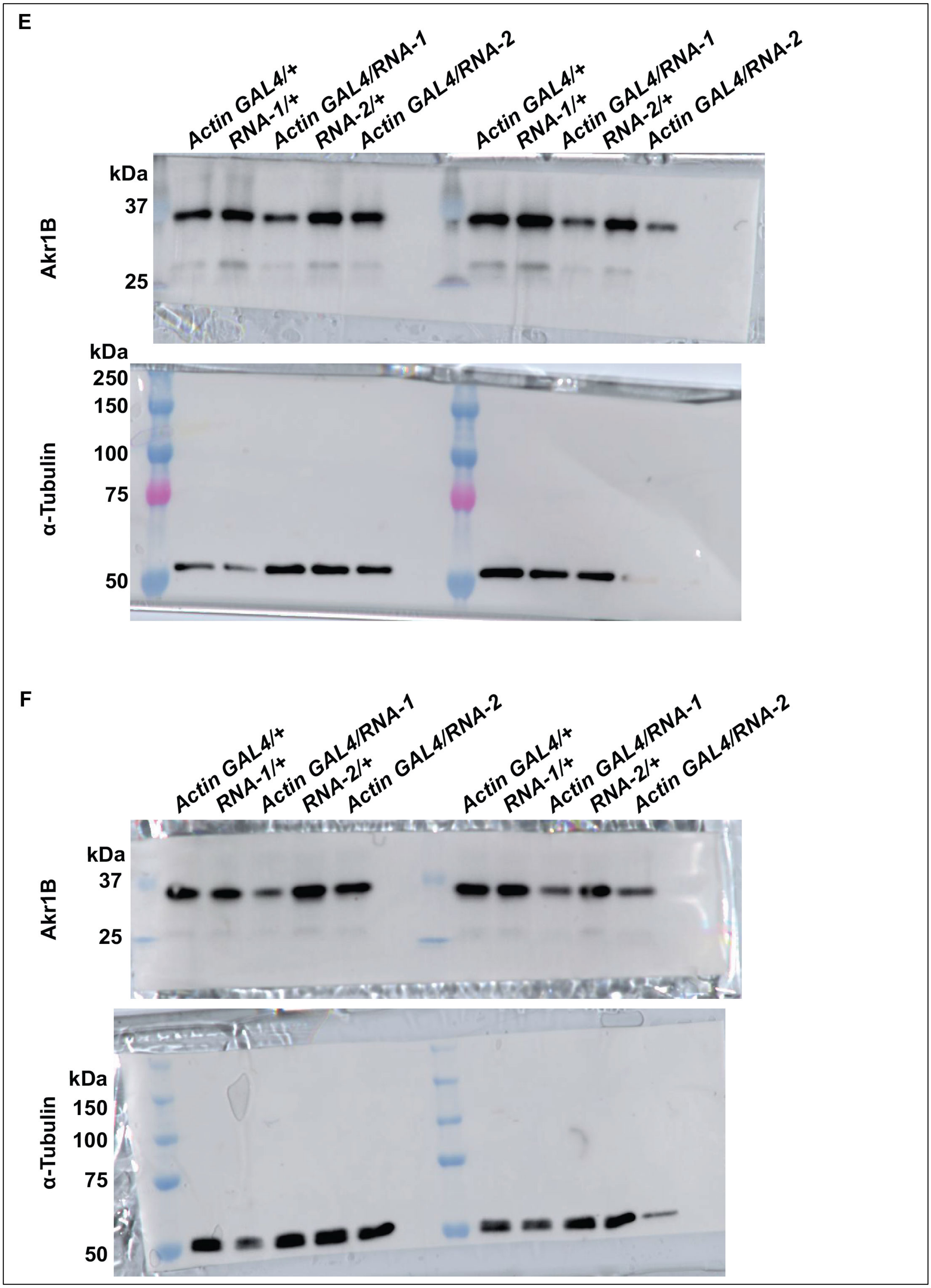
E
Actin GAL4/+
RNA-1/+
Actin GAL4/RNA-1
RNA-2/+
Actin GAL4/RNA-2
Actin GAL4/+
RNA-1/+
Actin GAL4/RNA-1
RNA-2/+
Actin GAL4/RNA-2
kDa
37
25
Akr1B
kDa
250
150
100
75
50
α-Tubulin
F
Actin GAL4/+
RNA-1/+
Actin GAL4/RNA-1
RNA-2/+
Actin GAL4/RNA-2
Actin GAL4/+
RNA-1/+
Actin GAL4/RNA-1
RNA-2/+
Actin GAL4/RNA-2
kDa
37
25
Akr1B
kDa
150
100
75
50
α-Tubulin

Table S1. Key Resources Table
Table of all the reagents and resources used throughout the manuscript.

Table S2. Genotype by figures
List of genotypes used in each figure.

Table S3. Raw data
Raw data used for quantifications in all the primary and supplemental figures.
